# The guided fire from within: intratumoral administration of mRNA-based vaccines to mobilize memory immunity and direct immune responses against pathogen to target solid tumors

**DOI:** 10.1038/s41421-024-00743-3

**Published:** 2025-01-02

**Authors:** Renhao Li, Jing-Chu Hu, Li Rong, Yige He, Xiaolei Wang, Xuansheng Lin, Wenjun Li, Yangfan Wu, Chaiyaporn Kuwentrai, Canhui Su, Thomas Yau, Ivan Fan-Ngai Hung, Xiang Gao, Jian-Dong Huang

**Affiliations:** 1 School of Biomedical Sciences, Li Ka Shing Faculty of Medicine, The University of Hong Kong, Pokfulam, Hong Kong SAR, China; 2 Department of Medicine, School of Clinical Medicine, Li Ka Shing Faculty of Medicine, The University of Hong Kong, Pokfulam, Hong Kong SAR, China; 3 Shenzhen Key Laboratory for Cancer Metastasis and Personalized Therapy Department of Clinical Oncology, The University of Hong Kong-Shenzhen Hospital, Shenzhen, Guangdong, China; 4 PGR Solutions Inc, Durham, NC USA; 5 Key Laboratory of Quantitative Synthetic Biology, Shenzhen Institute of Synthetic Biology, Shenzhen Institutes of Advanced Technology, Chinese Academy of Sciences, Shenzhen, Guangdong, China; 6 Guangdong-Hong Kong Joint Laboratory for RNA Medicine, Sun Yat-sen University, Guangzhou, China; 7 Materials Innovation Institute for Life Sciences and Energy (MILES), HKU-SIRI, Shenzhen, Guangdong, China

**Keywords:** Cancer immunotherapy, Tumour immunology, Cancer microenvironment

## Abstract

We investigated a novel cancer immunotherapy strategy that effectively suppresses tumor growth in multiple solid tumor models and significantly extends the lifespan of tumor-bearing mice by introducing pathogen antigens into tumors via mRNA-lipid nanoparticles. The pre-existing immunity against the pathogen antigen can significantly enhance the efficacy of this approach. In mice previously immunized with BNT162b2, an mRNA-based COVID-19 vaccine encoding the spike protein of the SARS-CoV-2 virus, intratumoral injections of the same vaccine efficiently tagged the tumor cells with mRNA-expressed spike protein. This action rapidly mobilized the pre-existing memory immunity against SARS-CoV-2 to kill the cancer cells displaying the spike protein, while concurrently reprogramming the tumor microenvironment (TME) by attracting immune cells. The partial elimination of tumor cells in a normalized TME further triggered extensive tumor antigen-specific T cell responses through antigen spreading, eventually resulting in potent and systemic tumor-targeting immune responses. Moreover, combining BNT162b2 treatment with anti-PD-L1 therapy yielded a more substantial therapeutic impact, even in “cold tumor” types that are typically less responsive to treatment. Given that the majority of the global population has acquired memory immunity against various pathogens through infection or vaccination, we believe that, in addition to utilizing the widely held immune memory against SARS-CoV-2 via COVID-19 vaccine, mRNA vaccines against other pathogens, such as Hepatitis B Virus (HBV), Common Human Coronaviruses (HCoVs), and the influenza virus, could be rapidly transitioned into clinical use and holds great promise in treating different types of cancer. The extensive selection of pathogen antigens expands therapeutic opportunities and may also overcome potential drug resistance.

## Introduction

Immunotherapy has emerged as a promising approach for treating cancers and has made great strides compared to traditional cancer therapy strategies. Despite significant progress in the past few decades, current cancer immunotherapies are still limited in terms of treating patients with different cancer types and stages. Effective cancer immunotherapies principally rely on the induction of the cancer immunity cycle^1^. The partial killing of cancer cells in the tumor by anti-tumor attack results in the further release of tumor antigens that induce more extensive and robust anti-tumor responses in the subsequent cancer immunity cycle^1^. However, tumors constantly evolve different mechanisms to escape immunosurveillance and disrupt the cancer immunity cycle, including downregulation of antigen presentation, establishment of immunosuppressive tumor microenvironment (TME), upregulation of immune checkpoints, etc., which ultimately results in the failure of the immunotherapy or the recurrence of the tumor^2,3^.

Novel strategies are needed to recover the effective cancer immune cycle. Direct intratumoral injection of the unadjuvanted seasonal influenza vaccine^4^, or drug-loaded oncolytic viruses^5,6^ was shown to reverse the immunosuppressive “cold” TME to a “hot” TME, and have potent anti-cancer effects. Our previous studies also demonstrated that the introduction of engineered tumor-targeted *Salmonella* in vivo could efficiently suppress tumor growth^7,8^ and tumor metastasis^9^. Alternatively, introducing foreign antigens into the tumor could make the tumor more recognizable to the immune system. Gary Nabel’s group used a direct intratumoral gene transfer approach to introduce an allogenic MHC molecule into tumor cells, resulting in rapid cell disruption mediated by the endogenous antibodies against the foreign MHC antigens. This was succeeded by strong T cell responses against tumor-specific antigens, which were obtained during the clearance of dead tumor cell debris through a process referred to as antigen spreading^10–12^. Additionally, loading tumor cells with influenza virus-derived MHC peptide ligands can also serve as a vaccine to induce more robust anti-tumor responses^13^. Moreover, synthetic peptides, mRNA, or DC cells-based neoantigen vaccines are currently being intensively researched as therapeutic options, aiming to train the immune system to recognize and eliminate solid tumors^14,15^.

However, the response rates to the cancer immunotherapies detailed above, as well as the clinically employed immunotherapy strategies, such as checkpoint blockade^16–19^ and CAR-T therapy^20–24^, remain limited when used alone. For enhanced therapeutic outcomes, it is important to concurrently regulate the TME and generate sufficient numbers of tumor-recognizing T cells.

In this study, we designed a novel immunotherapeutic strategy against cancer. First, we established memory immunity against pathogens in the host via regular vaccination routes. Next, we labeled tumor cells with pathogen antigens through intratumoral injections of antigen-encoding mRNA-lipid nanoparticles. The combination of pre-existing systemic active immunity against the pathogen antigen and the labeled tumor cells quickly initiated the influx of immune cells and the killing of tumor cells. This influx of a large number of active immune cells rapidly established a proinflammatory TME, effectively overcoming the immunosuppressive TME by converting a “cold tumor” into a “hot tumor,” thereby initiating an effective cancer immunity cycle.

We first tested the mRNA-based COVID-19 vaccine BNT162b2 (Comirnaty), developed by Pfizer-BioNTech, which encodes the pathogen antigen spike protein of SARS-CoV-2. This vaccine has been widely administered worldwide during the COVID-19 pandemic to effectively prevent severe SARS-CoV-2 infection. The BNT162b2 vaccine comprises spike protein mRNA encapsulated in lipid nanoparticles^25^ and works by provoking potent humoral and cellular immune responses against the spike protein^26–28^. By now, the majority of the global population should have acquired the immune memory against the spike protein of SARS-CoV-2, either through vaccination or by viral infection^29^.

We provided clear evidence that intratumoral administrations of the BNT162b2 vaccine in mice that had acquired anti-spike immune memory rapidly elicited strong T cell immune responses. These responses not only targeted the spike protein expressed in the tumor but also targeted the tumor antigens due to antigen spreading. The presence of pre-existing immunity against the spike protein, local inflammatory responses, reprogramming of the immune suppressive TME, and elevated antigen presentation activity were all prerequisites for more rapid, potent, and extensive tumor-targeting T cell responses. This immunotherapeutic strategy demonstrated broad-spectrum efficacy against several types of solid tumors in mice. It effectively inhibited tumor growth in treated lesions, and extended the lifespan of tumor-bearing mice. It also showed a systemic effectiveness by inhibiting the growth of untreated distal lesions in the same mouse, and significantly reduced the incidence of lung metastasis. Furthermore, following intratumoral injections of BNT162b2, we observed abundant tumor-infiltrating lymphocytes (TILs) with significantly increased local levels of proinflammatory markers, potent anti-tumor immune responses, and significantly increased PD-L1 expression on tumor-associated immune cells — hallmarks of a “hot tumor”^30^. The high PD-L1 expression prompted us to evaluate the therapeutic efficacy of BNT162b2 in combination with anti-PD-L1 treatment. The combined PD-L1 immune checkpoint inhibitor (ICI) treatment significantly potentiated the anti-tumor effect of this BNT162b2-based cancer therapeutic strategy, and in some cases, tumor nodules were eliminated by the combined therapy. Additionally, we demonstrated that intratumoral injections of mRNA vaccines against two other common viral pathogen antigens — the Large Hepatitis B surface antigen (L-HBsAg) and the spike protein of the Common Human Coronaviruses HKU1 (HKU1-S) — had similar anti-tumor activities against B16F10 melanoma in mice with immune memory against the same pathogen antigens. Importantly, the local treatment of solid tumors with these therapeutic vaccines did not cause apparent side effects or body weight changes, other than local inflammation, suggesting that the treatment is safe.

Together, we present a novel strategy to repurpose a readily available and clinically proven safe and effective COVID-19 mRNA vaccine as a powerful tool to treat cancer. The robust safety and therapeutic efficacy data across various cancer types provide a strong foundation for further translational studies, paving the way to bring this approach to the clinic. Moreover, the mRNA vaccines encoding other pathogen antigens also showed potent antitumor effects in hosts with pre-existing immunity, suggesting that our proposed therapeutic strategy could have broad implications for vaccine choice. This could offer more treatment options for cancer patients based on their vaccination or infection history, which is potentially significant for clinical application.

## Results

### Intratumoral injections of BNT162b2 demonstrates strong therapeutic efficacy against multiple types of solid tumors in mice that had the pre-existing immunity against SARS-CoV-2 spike protein

**Fig. 1:**
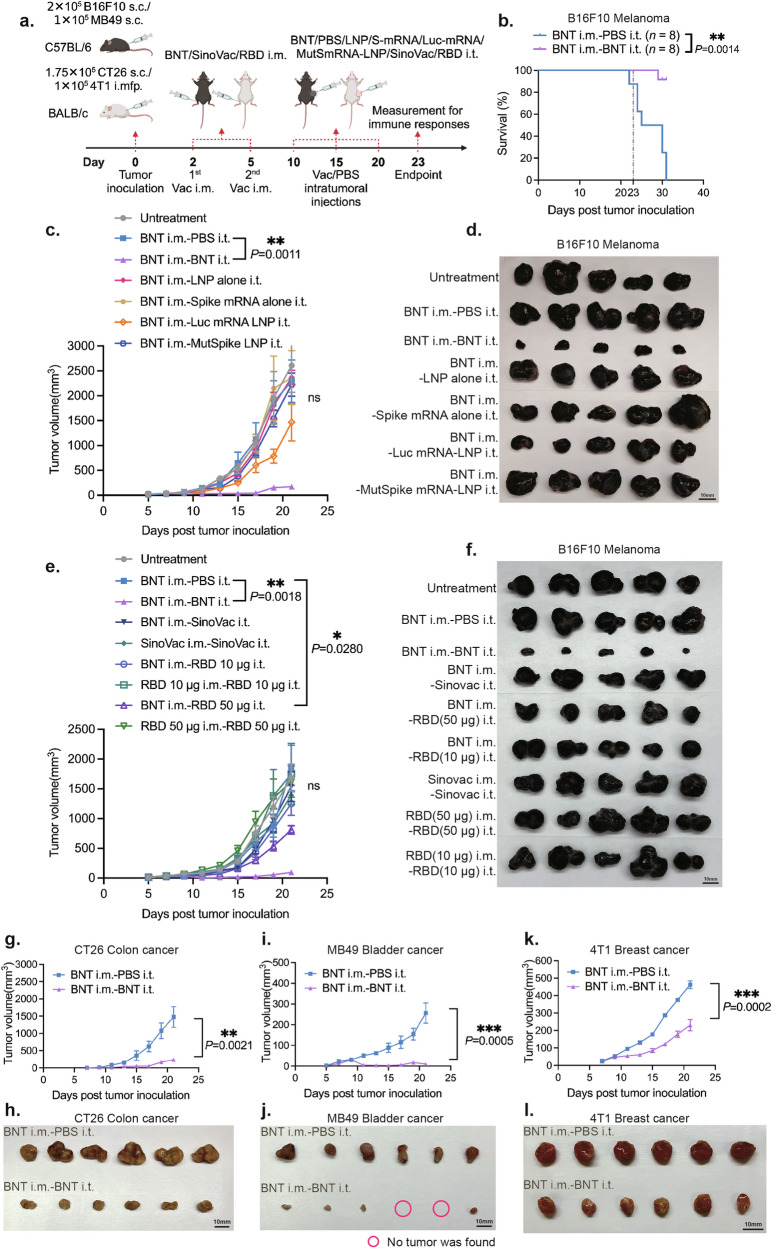
Intratumoral injection of BNT162b2 reduces tumor growth and extends survival in vaccinated mice. **a** Experimental design of the BNT162b2-based cancer therapy. Intramuscular BNT162b2 vaccinations were administered after cancer cell implantation (*n* = 5 or 6 per group). **b** Survival curves comparing intratumoral BNT162b2 treatment group and intratumoral PBS control group. **c**, **e** Tumor growth curves of the intratumoral BNT162b2 treatment group vs untreatment control group, PBS control group, LNP alone control group, spike mRNA alone control group, Luc mRNA-LNP control group, mutant spike mRNA-LNP control group, SinoVac control groups, and RBD control groups in the B16F10 melanoma model. The experiments are described in **a**. **g**, **i**, **k** Tumor growth curves of the intratumoral BNT162b2 treatment group vs PBS control group in the CT26 colon cancer model, MB49 bladder cancer model, and 4T1 breast cancer model, based on the experimental design in **a**. **d**, **f**, **h**, **j**, **l** Photos of tumors from the treatment and control groups at the endpoints of the experiments described in **a**. i.m.: intramuscular injection; i.t.: intratumoral injection; s.c.: subcutaneous injection; i.mfp.: intramammary fat pad injection.

To test whether BNT162b2 can be repurposed as a broad-spectrum and potent anticancer therapeutic agent against solid tumors, B16F10 murine melanoma tumor was established in mice. We tested two treatment schemes with different orders of vaccination. In the first scheme, all the mice received two doses of intramuscular vaccinations of 50 μL diluted BNT162b2 (containing 5 μg mRNA) on Days –42 and –21 prior to subcutaneous implantation of 3 × 10^5^ B16F10 melanoma cells. On Days 5, 10, and 17 after tumor inoculation, we performed intratumoral injections of 50 μL diluted BNT162b2 in the treatment group or 50 μL PBS in the control group (Supplementary Fig. S1a). In the second scheme, mice were first inoculated with 2 × 10^5^ B16F10 cells subcutaneously. On Days 2 and 5 after tumor inoculation, mice were vaccinated intramuscularly with 50 μL diluted BNT162b2. On Days 10, 15, and 20 after tumor inoculation, mice received intratumoral injections of 50 μL diluted BNT162b2 or PBS (Fig. 1a). We observed profound inhibition of tumor growth in both treatment groups that received intratumoral injections of BNT162b2 compared to the control group that received intratumoral injections of PBS, regardless of whether the intramuscular vaccination was performed prior to tumor inoculation (Supplementary Fig. S1b–d) or post tumor inoculation (Fig. 1c, e). This was also supported by a reduction in tumor size at the endpoint (Fig. 1d, f; Supplementary Fig. S1e) and by a marked improvement in the survival rate of mice bearing melanoma (Fig. 1b). To determine whether pre-established immunity against pathogen antigens is crucial, we conducted several control experiments in which intratumoral injections of BNT162b2 were replaced with intratumoral injections of lipid nanoparticles (LNP) alone (empty LNP with the same lipid composition as that of BNT162b2 without encapsulating spike mRNA), spike mRNA alone (the mRNA same as that of BNT162b2 without LNP encapsulation), Luciferase mRNA-LNP (Luc mRNA-LNP, where Luciferase mRNA was encapsulated in the same LNP as BNT162b2), or mutant spike mRNA-LNP (with all possible start codons replaced by stop codons and encapsulated in the same LNP as BNT162b2) (Fig. 1c, d). Among these control groups, the therapeutic efficacy either disappeared completely or had a minimal effect (as observed in the Luc mRNA-LNP intratumoral injection group) (Fig. 1c, d). Although the intratumoral injections of Luc mRNA-LNP also introduce the foreign antigen into the tumor, it differs from the BNT162b2 treatment group; the absence of systemic memory immunity prevented a rapid activation of Luciferase-specific T cell responses, unlike the BNT162b2 treatment group with memory immunity against the SARS-CoV-2 spike protein. To determine whether intracellular expression of pathogen antigens via mRNA vaccine is crucial, we compared the therapeutic efficacy of intratumoral injections of BNT162b2 with that of intratumoral injections of the inactivated COVID-19 vaccine (SinoVac) or the RBD protein in previously intramuscularly vaccinated mice with BNT162b2 (Fig. 1e, f). Our data suggest that intratumoral injections of RBD protein demonstrate some therapeutic efficacy, but they are still not as effective as the BNT162b2 treatment group, while the therapeutic efficacy of the group that received intratumoral injections of SinoVac is quite limited (Fig. 1e, f). We believe that both RBD and SinoVac intratumoral injections can recruit spike-specific memory T cells into the tumor. However, unlike BNT162b2 intratumoral injections, RBD or SinoVac intratumoral injections do not tag tumor cells with spike or RBD proteins, and therefore do not direct memory T cells to specifically target and kill tumor cells.

Additionally, considering that a portion of people worldwide have obtained SARS-CoV-2-specific memory immunity through inactive virus vaccine administrations, we also investigated whether the BNT162b2-based cancer therapy remains effective for these individuals. Our data suggest that the BNT162b2 intratumoral injections can inhibit tumor growth in mice that have acquired anti-SARS-CoV-2 immunity through SinoVac intramuscular vaccination. However, its therapeutic efficacy is not as potent as in individuals who received the BNT162b2 intramuscular vaccine (Supplementary Fig. S1i–k). This can be attributed to the lower T cell response against the spike protein induced by SinoVac compared to BNT162b2^31^. In conclusion, these results not only demonstrate the therapeutic efficacy of BNT162b2-based cancer therapy but also highlight the advantages of mRNA vaccines in our cancer therapy strategy compared to other types of COVID-19 vaccines.

In addition, we observed that prior intramuscular BNT162b2 vaccination followed by intratumoral injections of BNT162b2 also had excellent therapeutic efficacy in the CT26 colon cancer model (Fig. 1g, h) and even greater therapeutic efficacy in the MB49 bladder cancer model (Fig. 1i, j). Two out of six MB49 bearing mice in the BNT162b2 treatment group showed total elimination of tumor mass (Fig. 1j). We also observed that BNT162b2 had a significant inhibitory effect on tumor growth at tumor implantation sites in the 4T1 breast cancer model (Fig. 1k, l), with a significantly reduced incidence of lung metastasis (Supplementary Fig. S1f–h). These data strongly suggest that intramuscular BNT162b2 vaccination followed by intratumoral BNT162b2 treatment has potent therapeutic efficacy in all four cancer types tested.

### BNT162b2-based cancer therapy enhances the intratumoral infiltration of immune cells, increases the MHC expression in tumor cells, and reverses the immunosuppressive TME

**Fig. 2:**
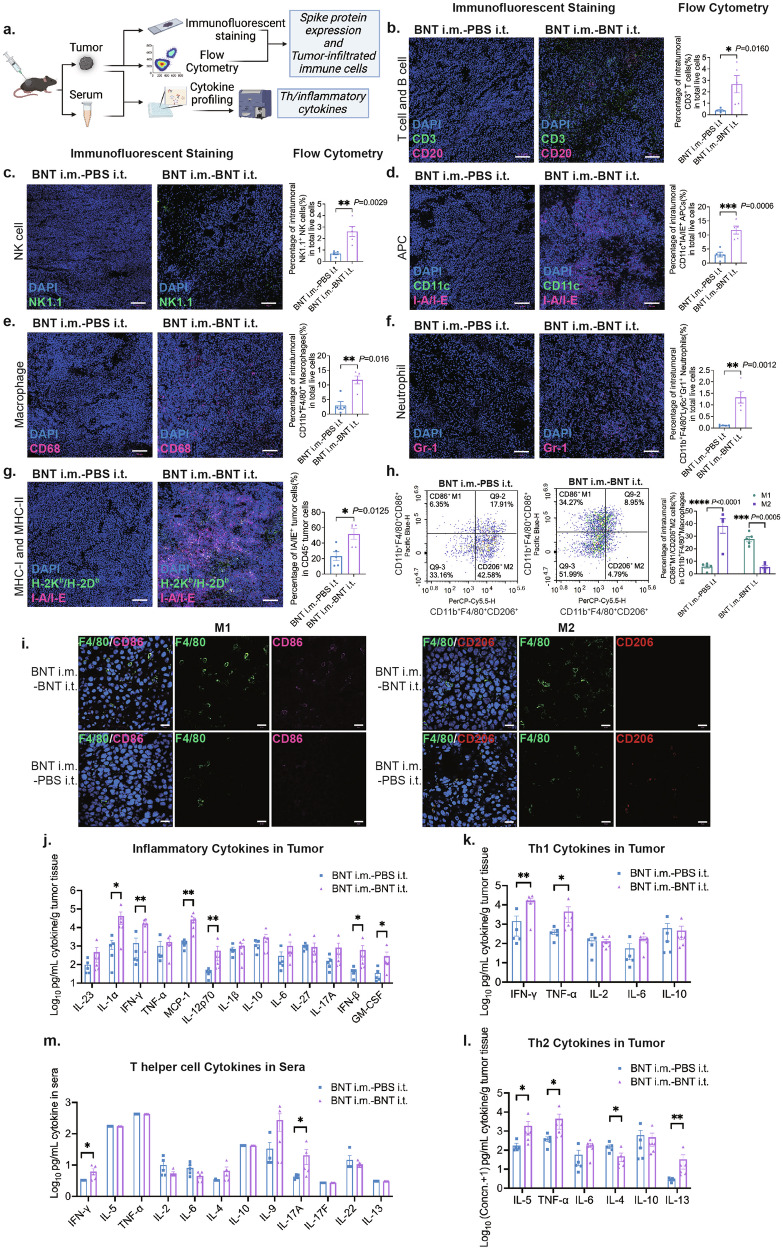
BNT162b2-base cancer immunotherapy induces strong immune cell recruitment and TME reprogramming. **a** Experimental design to characterize immune responses following BNT162b2-based cancer therapy. **b**–**f** Representative immunofluorescent staining images of tumor-infiltrating immune cells, including CD3^+^ T lymphocytes, CD20^+^ B lymphocytes, NK1.1^+^ NK cells, CD11c^+^ I-A/I-E^+^ APCs, CD68^+^ macrophages, and Gr-1^+^ neutrophils. Please zoom in to see the fluorescent signals. And quantitative flow cytometry analysis (*n* = 5 per group) of CD3^+^ T lymphocytes, NK1.1^+^ NK cells, CD11c^+^ I-A/I-E^+^ APCs, CD11b^+^ F4/80^+^ macrophages, and CD11b^+^ F4/80^*−*^Ly6C^+^ Gr1^+^ neutrophils. **g** Representative immunofluorescent staining images and quantitative flow cytometry analysis (*n* = 5 per group) of H2K^b^/H2D^b^ and I-A/I-E in the tumor. (Please zoom in to see the fluorescent signals). **h** Detection of intratumoral macrophage phenotypes M1 and M2 by flow cytometry (*n* = 5 per group). **i** Representative immunofluorescent staining images of tumor-infiltrating F4/80^+^ CD86^+^ M1 cells and F4/80^+^ CD206^+^ M2 cells in treatment and control groups. **j** Inflammatory-associated cytokine profiling of tumor tissues (*n* = 5 in BNT i.m.-PBS i.t. group, *n* = 6 in BNT i.m.-BNT i.t. group). **k**, **l** T helper cell-associated cytokine profiling of tumor tissues (*n* = 5 in BNT i.m.-PBS i.t. group, *n* = 6 in BNT i.m.-BNT i.t. group). **m** T helper cell-associated cytokine profiling of sera (*n* = 5 in BNT i.m.-PBS i.t. group, *n* = 5 in BNT i.m.-BNT i.t. group). BNT i.m.-PBS i.t. (control group): the mice with BNT162b2 intramuscular injections and PBS intratumoral injections. BNT i.m.-BNT i.t. (treatment group): the mice with BNT162b2 intramuscular injections and BNT162b2 intratumoral injections. Scale bars, 100 μm in **b**–**g**, and 20 μm in **i**. *P < 0.05; **P < 0.01 in **j**–**m**.

To detect the tumor-infiltrating immune cells, tumor sections from different treatment groups were assayed by immunofluorescent staining, and cell suspensions from tumor tissues were prepared by collagenase digestion and analyzed by flow cytometry (Fig. 2a). We observed a larger percentage of CD45^+^ leukocytes in tumors post BNT162b2 intratumoral injections compared to in PBS-intratumoral treated tumors (Supplementary Fig. S2b). We also observed significantly elevated percentages of tumor-infiltrating T lymphocytes, macrophages, NK cells, antigen-presenting cells (APCs), and neutrophils and an increased trend of B cells in the BNT162b2 treatment group compared to the control group. Specifically, we identified higher levels of CD3^+^ T cells, CD20^+^ B cells, NK1.1^+^ NK cells, CD11c^+^I-A/I-E^+^ APCs, CD68^+^ macrophages, and Gr-1^+^ neutrophils by immunofluorescent staining on tumor sections from mice post BNT162b2 intratumoral injection, which was consistent with the flow cytometry data (Fig. 2b–f). Besides, we observed a trend of increasing percentage of CD19^+^ B cells by flow cytometry analysis, but this did not reach statistical significance when compared to the control group (Supplementary Fig. S2c). The expression levels of both MHC-I (H-2K^b^/H-2D^b^) and MHC-II (I-A/I-E) were significantly increased in the stained tumor sections (Fig. 2g) from mice received the BNT162b2 intratumoral injections, which was supported by a significant increase in the MHC-II (I-A/I-E)-positive population in CD45^–^ tumor cells detected by flow cytometry (Fig. 2g). These results suggest that intratumoral BNT162b2 treatment significantly increased the recruitment of immune cells into the tumor mass and enhanced the expression of the MHC molecules on both tumor cells and APCs, which are critical for the tumor antigen presentation and the subsequent anti-tumor immune responses. Simultaneously, our data also showed increased expression levels of heat shock proteins (HSPs), Calreticulin and HSP70, in the tumor cells post BNT162b2 treatments (Supplementary Fig. S2d, e). Intracellular HSPs act as chaperones of tumor antigen-derived peptides and have been implicated in the transfer and presentation of tumor antigens^32^.

In addition to promoting the infiltration of immune cells in tumors, BNT162b2 treatment also reversed the immunosuppressive TME to an immunoactive TME that is in favor of anti-tumor immunity. Compared to the control group, the intratumoral BNT162b2 treatment led to a significant increase in the number of intratumoral F4/80^+^ macrophages (Fig. 2e, i), an increased percentage of intratumoral CD86^+^ M1 macrophages, and a decreased percentage of intratumoral CD206^+^ M2 macrophages (Fig. 2h, i). This suggests a reversal of intratumoral macrophage phenotype from M2 to M1. Furthermore, we observed a pro-inflammatory cytokine profile that was more supportive of T cell immune responses, with increased levels of intratumoral proinflammatory cytokines IFN-γ, TNF-α, IFN-β, IL-1α, MCP-1, IL-12p70, and GM-CSF (Fig. 2a, j). We also found significantly increased levels of intratumoral cytokines IFN-γ, TNF-α, and IL-5, known to be T helper 1 (Th1) and T helper 2 (Th2)-related cytokines that play a role in anti-tumor immune responses (Fig. 2a, k, l). IL-5 is involved in stimulating B lymphocytes to produce antibodies^33,34^, which was consistent with an increase in the titer of anti-spike antibodies post BNT162b2-based cancer therapy (Fig. 4i)^35^. However, other Th9, Th17, or Th22-associated cytokines were not significantly elevated in the tumor samples after the BNT162b2-based cancer therapy (Fig. 2a; Supplementary Fig. S3a–c). Notably, the cytokine profiles in peripheral blood also showed increased systemic IFN-γ levels (Fig. 2a, m).

The release of cytokines in the tumor and peripheral blood indicates that the BNT162b2-based cancer therapy can effectively induce an anti-tumor response and reverse the immunosuppressive TME to an anti-tumor phenotype. Although most cytokines changed in the tumor induced by BNT162b2 play a role in tumor-killing, there was a significant decrease in intratumoral IL-4, and significant increases in intratumoral IL-13 and peripheral IL-17A (Fig. 2l, m). The effects of IL-4, IL-13, and IL-17A on tumor immunity are controversial, as various studies have shown they are associated with tumor progression or tumor suppression^34–38^.

Taken together, BNT162b2-based cancer therapy induced the recruitment of various immune cells into the tumor, reversed the immunosuppressive TME to an anti-tumor phenotype, and increased intratumoral or systemic Th1, Th2, and inflammatory-related cytokines, leading to positive therapeutic effects.

### Transcriptome analysis reveals activated T cells under a complex immune environment

**Fig. 3:**
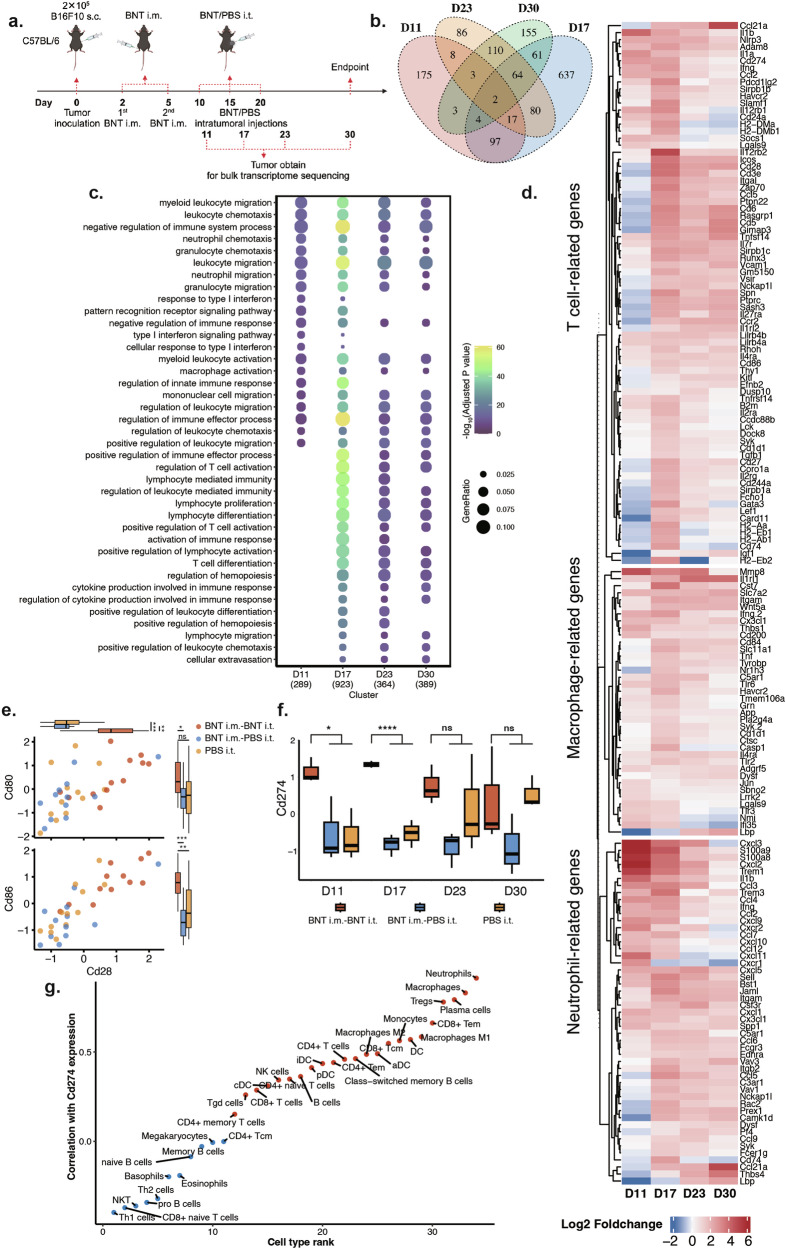
Transcriptome profile changes post intratumoral BNT162b2 injections. **a** Schematic diagram of the experimental design of the RNA-seq. C57BL/6 mice were vaccinated with BNT162b2 via intramuscular injection on Days 2 and 5 after tumor inoculation; intratumoral BNT162b2 injections or PBS injections were performed on Days 10, 15, and 20. Tumor tissues were collected on Days 11, 17, 23, and 30 from mice in the BNT i.m.-BNT i.t. treatment group, as well as the BNT i.m.-PBS i.t. and PBS i.t. (mice only got PBS intratumoral injections) control groups, respectively. *n* = 3 each timepoint for each group. **b** Venn plot comparing the differential gene expression between intratumoral BNT162b2 treatment group (BNT i.m.-BNT i.t. group) and the two control groups (BNT i.m.-PBS i.t. group and PBS i.t. only group) at different time points. **c** Enriched GO terms of significantly upregulated genes after BNT162b2 intratumoral injections. **d** Heatmap showing the fold change of activation genes in the neutrophil, macrophage and T cell after BNT162b2 intratumoral injections. **e** The correlation between APC markers and T cell activation markers. Wilcoxon test was used to analyze statistical differences. **f** Dynamic expression of PD-L1 (CD274) during BNT162b2-based cancer therapy. Statistical differences between the BNT162b2 intratumoral injection group (BNT i.m.-BNT i.t. group) and the other two control groups (BNT i.m.-PBS i.t. group and PBS i.t. only group) were determined by DESeq2. **g** The correlation between the PD-L1 expression and the activation score of different cell types. BNT i.m.-PBS i.t. (control group-1): the mice with BNT162b2 intramuscular injections and PBS intratumoral injections. PBS i.t. (control group-2): the mice with only PBS intratumoral injections. BNT i.m.-BNT i.t. (treatment group): the mice with BNT162b2 intramuscular injections and BNT162b2 intratumoral injections. *P < 0.05; **P < 0.01; ***P < 0.001; ****P < 0.0001. ns not statistically significant.

To analyze the changes in immune status in the TME during the BNT162b2-based cancer therapy, we collected tumor tissue samples at different time points for bulk RNA-seq analysis (Fig. 3a). Our results showed that the gene expression profile of the tumor tissues was altered compared to the control groups following both the first and second doses of intratumoral BNT162b2 injections (Supplementary Fig. S4a). The differential gene expression profile between the intratumoral BNT162b2 treatment group (BNT i.m.-BNT i.t. group) and the control groups (PBS i.t. group and BNT i.m.-PBS i.t. group) at different time points showed little overlap (Fig. 3b), suggesting dynamic and significant changes occurred in the TME during the BNT162b2-based cancer therapy. The Gene Ontology (GO) enrichment analysis revealed that myeloid leukocytes including neutrophils and macrophages were activated throughout the course of intratumoral BNT162b2 treatment (Fig. 3c). This is consistent with the inferred activation score in the immune cell type analysis (Supplementary Fig. S4b), possibly associated with the anti-tumor innate immune responses. The expressions of IFNγ (Fig. 3d) and pattern recognition receptor signaling pathways (Fig. 3c) were also significantly upregulated or enriched following the intratumoral BNT162b2 injections, which is similar to the mRNA vaccine studies reported by Arunachalam et al.^39^. In addition to the activation of innate immune responses, T cell activation was increased throughout the course of the intratumoral BNT162b2 treatment, except the day after the first intratumoral injection (Fig. 3c, d; Supplementary Fig. S4b). A significant upregulation with a high degree of correlation in the gene expression of CD28 and B7 family members (CD80, CD86) was observed, suggesting that co-stimulatory signals from APCs could be involved in T cell activation (Fig. 3e)^40^.

Interestingly, we observed a significant upregulation of CD274 (PD-L1) during the first three time points after the intratumoral BNT162b2 injections (Fig. 3f). It has been reported that tumor-associated neutrophils and macrophages negatively regulate the adaptive immunity through an increased expression of PD-L1 in different tumor types^41–43^. Our analysis also suggested that PD-L1 expression was strongly correlated with the activation of neutrophils and macrophages (Fig. 3g).

The transcriptome analysis following the intratumoral BNT162b2 injections demonstrated that several cell types critical for adaptive and innate anti-tumor immunity were rapidly activated in the TME, including T cells, macrophages, and neutrophils.

### The effects of the BNT162b2-based cancer therapy depends on T cell response and induces potent tumor antigen spreading

**Fig. 4:**
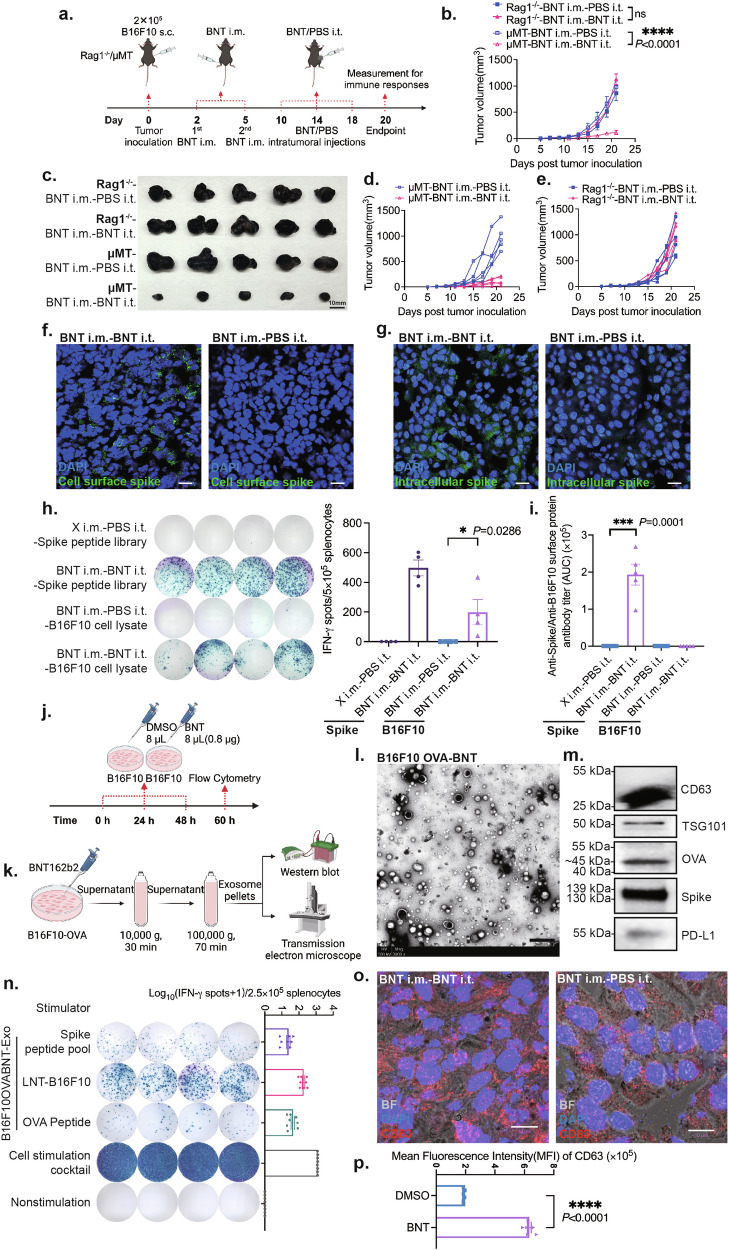
BNT162b2-based cancer therapy depends on T cell responses but not B cell responses, and induces potent antigen spreading by tumor cell killing and the release of tumor-derived exosomes. **a** Experimental design for investigating the role of B and T cells in BNT162b2-based cancer therapy in μMT or Rag1^*−/−*^ mice (*n* = 5 per group). **b** Tumor growth curves of the intratumoral BNT162b2 treatment group vs the intratumoral PBS control group for the experiment described in **a**. **c** Photo of tumors at the endpoint in the treatment and control groups from the experiment described in **a**. **d** Tumor growth curves of the intratumoral BNT162b2 treatment group vs the intratumoral PBS control group in μMT mice. **e** Tumor growth curves of the intratumoral BNT162b2 treatment group vs the PBS control group in Rag1^*−/−*^ mice. **f** Expression of SARS-CoV-2 spike protein on the surface of tumor cells. **g** Expression of SARS-CoV-2 spike protein inside tumor cells. **h** Representative IFN-γ ELISpot assays show tumor antigen-specific T cell responses post intratumoral BNT162b2 therapy (*n* = 4 per group). **i** Titers of anti-spike protein and anti-B16F10 surface protein IgG antibody in sera post intratumoral BNT162b2 injections or PBS injections (*n* = 5 per group). In **h** and **i**, X i.m.*-*PBS i.t.: sera or splenocytes from mice without any intramuscular vaccination but with intratumoral PBS injections. BNT i.m.*-*BNT i.t.: sera or splenocytes from mice with intramuscular and intratumoral BNT162b2 injections. BNT i.m.-PBS i.t.: sera or splenocytes from mice with intramuscular BNT162b2 injections and intratumoral PBS injections. **j** Experimental design of the detection of intracellular CD63^+^ exosomes in the B16F10 cell line post BNT162b2 treatments or DMSO treatments (*n* = 5 per group). **k** Experimental design for investigating the characteristics of exosomes derived from BNT162b2 treated B16F10-OVA cells. **l** Transmission electron microscopy (TEM) images of isolated B16F10-OVA-derived exosomes. **m** Identification of B16F10-OVA-derived exosomes by western blotting assay, and detecting the expression of spike protein, OVA protein, and PD-L1 in B16F10-OVA-derived exosomes. **n** Representative IFN-γ ELISpot assays show tumor antigen-specific T cell responses induced by BNT162b2 transfected B16F10-OVA-derived exosomes (*n* = 8 per group, full data are shown in Supplementary Fig. S6e). **o** Immunofluorescent staining of CD63 (marker of exosomes) on tumor sections from mice described in Fig. 1a. **p** Comparing the mean fluorescence intensity (MFI) of intracellular CD63^+^ exosomes in B16F10 cells treated with BNT162b2 to that in B16F10 cells treated with DMSO, as determined by flow cytometry. The experimental procedure is described in **j**. BNT i.m.-PBS i.t. (control group): the mice with BNT162b2 intramuscular injections and PBS intratumoral injections. BNT i.m.-BNT i.t. (treatment group): the mice with BNT162b2 intramuscular injections and BNT162b2 intratumoral injections. Scale bars, 20 μm in **f** and **g**, 1 μm in **l**, and 10 μm in **o**. *P < 0.05; **P < 0.01; ***P < 0.001; ****P < 0.0001. ns, not statistically significant.

To further study the mechanisms underlying the BNT162b2-based cancer therapy, we investigated the specific contributions of B cell and T cell responses in B cell- or T cell-deficient mice. For B cell responses, we assessed the efficacy of BNT162b2-based cancer therapy in μMT mice that lack mature B lymphocytes^44^. For T cell responses, we assessed the efficacy of BNT162b2-based cancer therapy in Rag1^*−/−*^ mice that have small lymphoid organs that do not produce mature B and T lymphocytes^45^. We established B16F10 melanoma subcutaneous models in μMT and Rag1^*−/−*^ mice followed by the intramuscular vaccinations and intertumoral BNT162b2 treatments (Fig. 4a). The results showed that the BNT162b2 treatments had therapeutic effects in the B16F10 melanoma model in μMT mice that were comparable to that in the C57BL/6 mice (Fig. 4b–d), but had no observed therapeutic effects in Rag1^*−/−*^ mice (Fig. 4b, c, e), which suggests that the anti-tumor effects of BNT162b2-based cancer therapy is predominantly dependent on T cell responses and less dependent on B cell responses.

Next, tumor, spleen, and serum samples were collected from tumor-bearing wild-type (WT) mice with intratumoral BNT162b2 or PBS treatment (Fig. 2a). The expression of the spike protein in the tumor tissue after intratumoral injections of BNT162b2 was confirmed by immunofluorescent staining of tumor sections, which showed very high levels of SARS-CoV-2 spike protein on the surface and in the cytoplasm of tumor cells (Fig. 4f, g) indicating that the intratumoral BNT162b2 treatment had successfully introduced the spike protein to tumor cells as an “artificial tumor antigen”.

We next explored if the anti-spike immunity was activated/reactivated to induce potent immune responses against spike protein-bearing tumor cells and effectively killed these tumor cells. We first validated whether the intratumoral BNT162b2 injection rapidly activated anti-spike T cell responses. Splenocytes were isolated from naive mice or the mice that received two doses of BNT162b2 intramuscular vaccinations. B16F10 cells were cocultured with these splenocytes simultaneously; the cell mixture was transfected with BNT162b2 to mimic the BNT162b2 intratumoral injections in vivo (Supplementary Fig. S5a). Twenty hours after the BNT162b2 transfection, T cell activation was detected by the expression of the T cell early activation marker CD69 and the IFN-γ secretion level of T cells. We found that T cells were activated more rapidly and profoundly in the group of B16F10 cocultured with splenocytes from vaccinated mice, compared with splenocytes from unvaccinated mice (Supplementary Fig. S5b, c). We also investigated whether nontargeted tumor antigens were recognized during the cleanup process of the dead tumor cells, and subsequently triggered immune responses against tumor-specific antigens (antigen spreading). We measured spike-specific or tumor antigen-specific T cell responses by ELISpots assays with splenocytes from different groups using spike protein peptides or the lysate from B16F10 cells without BNT162b2 treatments as the stimulating agent, respectively. The results showed a potent spike protein-specific T cell activation in the mice that received intramuscular injections of BNT162b2 (Fig. 4h). Furthermore, potent T cell responses against B16F10 cell lysate were also detected in splenocytes from mice that received intratumoral injections of BNT162b2, while the T cell response was extremely weak in PBS-intratumoral treated mice, indicating that the tumor antigen-specific T cell response was induced by the intratumoral injections of BNT162b2 (Fig. 4h). This evidence suggests that intratumoral injection of BNT162b2 can provoke broad tumor-specific anti-tumor T cell responses in hosts through a rapid mobilization of the established immunity against the spike protein.

To assess the antibody responses induced by BNT162b2-based cancer therapy, we performed enzyme-linked immunosorbent assay (ELISA) assays to measure serum antibody titers against SARS-CoV-2 spike protein and tumor antigens in mice from different groups. The results showed a significant increase in spike protein-specific antibody titer after BNT162b2-based cancer therapy (Fig. 4i). However, we did not detect the presence of any measurable antibodies against tumor surface proteins in the BNT162b2 treatment or the PBS control groups (Fig. 4i), which is consistent with the therapy results obtained with the μMT-deficient mice; hence, tumor-specific antibody responses may not be a key mechanism involved in BNT162b2-based cancer therapy (Fig. 4b–d, and i). In addition to the B16F10 melanoma model, we also validated the induction of tumor-specific T cell responses in the MB49 bladder cancer model after BNT162b2-based cancer therapy. The ELISpot results suggest that the specific T cell response against MB49 neoantigens was significantly increased in the intratumoral BNT162b2 treatment group compared to the PBS control group (Supplementary Fig. S5d–f). Furthermore, we validated that the antigen spreading induced by BNT162b2-based cancer therapy was systemic in a bilateral tumor model (Supplementary Fig. S5g). We implanted the B16F10 tumor on both left and right flanks of the mice, only the tumor nodules on the right flank were intratumorally injected with BNT162b2, and the tumor sizes on both flanks were measured. The tumor growth inhibition was noted for nodules on both flanks of the mice, which suggested that the tumor antigen-specific immune responses triggered by the BNT162b2 injections on the right flank were systemic and capable of inhibiting the growth of the tumor on the distal untreated flank of the mice (Supplementary Fig. S5h). This result indicated the potential application of the BNT162b2-based cancer therapy in cancer patients with multiple tumor metastases.

Studies have shown that tumor-derived exosomes carrying tumor antigens can induce tumor-specific T cell responses^46^. In addition, circulating exosomes with SARS-CoV-2 spike protein were detected after SARS-CoV-2 mRNA vaccination^47^. These observations prompted us to hypothesize that intratumoral vaccination with BNT162b2 can stimulate the release of exosomes containing not only the SARS-CoV-2 spike protein but also tumor-specific antigens from treated tumor cells, which may contribute to antigen spreading that triggers tumor-specific T cell responses. On the other hand, we also hypothesized that exosomes with the spike protein would have increased immunogenicity over those exosomes only containing tumor antigens, which could in turn results in more efficient tumor antigen presentation, leading to more potent tumor suppression. To test whether intratumoral BNT162b2 injections stimulate the release of exosomes carrying the spike protein together with tumor antigens, and that the released exosomes are involved in the activation of tumor antigen-specific T cell responses, we transfected B16F10-OVA tumor cells (a B16F10 cell line with stable expression of chicken ovalbumin) with BNT162b2. The supernatant of the transfected B16F10-OVA culture medium was collected, and exosomes were isolated (Fig. 4k), which showed typical sizes and morphology of exosomes under transmission electron microscopy (TEM) (Fig. 4l). The western blotting analysis showed that the isolated exosomes from BNT162b2-transfected tumor cells carried not only exosome markers CD63 and TSG101, but also the SARS-CoV-2 spike protein and OVA protein (Fig. 4m).

We next explored whether the exosomes carrying the spike protein and tumor antigens from BNT162b2-transfected B16F10-OVA cells could potentially induce tumor-specific T cell responses. We used liquid nitrogen treated B16F10 cells (LNT-B16F10), OVA-MHCI/-MHCII epitope peptides (25-amino acid length OVA peptides containing either OVA-MHCI or OVA-MHCII epitope, the sequence information is listed in Methods and Materials) and the peptide pool of the spike protein as stimulants to detect spike protein-specific and tumor antigen-specific T cell responses in the spleen from mice that had been subcutaneously injected with exosomes, respectively (Supplementary Fig. S6d). Fixable viability dye was used to validate that all B16F10 cells were dead after the liquid nitrogen treatment (Supplementary Fig. S6a). The ELISpot results of the immunogenicity of BNT162b2-transfected B16F10-OVA-derived exosomes indicated that they could induce SARS-CoV-2 spike protein-specific immune responses, and also tumor antigen- and OVA-specific immune responses (Fig. 4n; Supplementary Fig. S6e). On the other hand, we tested whether tumor antigen spreading could be induced by dead tumor cells. We subcutaneously immunized the mice with LNT-B16F10 (Supplementary Fig. S6b). As indicated in the ELISpot assay result with LNT-B16F10 as a stimulant, tumor antigen spreading did occur via dead tumor cells (Supplementary Fig. S6c). Simultaneously, BNT162b2 treatments led to increasing release of tumor antigen-contained exosomes from cultured cells in vitro (Fig. 4j, m, p) and from tumors grown in vivo (Fig. 4o). The level of exosome secretion was evaluated by staining for intracellularly located CD63. CD63 is a marker of unsecreted exosomes in late endosomal organelles, which has been used as a tool to track exosome secretion^48^. Taken together, the BNT162b2-based cancer therapy potentially increased the tumor antigen spreading by enhancing tumor antigen-contained exosome release and tumor cell death via effective tumor-killing. However, we were unable to observe any significant increase of immunogenicity in BNT162b2-transfected LNT-B16F10 or spike protein-containing exosomes compared with untransfected LNT-B16F10 or exosomes derived from untransfected B16F10, suggesting that the spike protein expression induced by BNT162b2 treatments is unable to directly increase antigen spreading by enhancing immunogenicity (Supplementary Fig. S6b–e).

These results are consistent with the idea that the BNT162b2-mediated cancer therapy is T cell response-dependent and could induce potent tumor antigen spreading through dead tumor cells and tumor cell-derived exosomes.

### PD-L1 expression in TME negatively affects the immunotherapeutic efficacy of BNT162b2, which can be overcome by the combinational therapy with anti-PD-L1

We showed that BNT162b2-based cancer therapy has excellent therapeutic efficacy in the B16F10 melanoma model, but the BNT162b2 treatment alone could not fully eradicate the tumor. In this regard, we further investigated the features of TME post BNT162b2 treatment to explore ways to enhance the therapeutic efficacy.

**Fig. 5:**
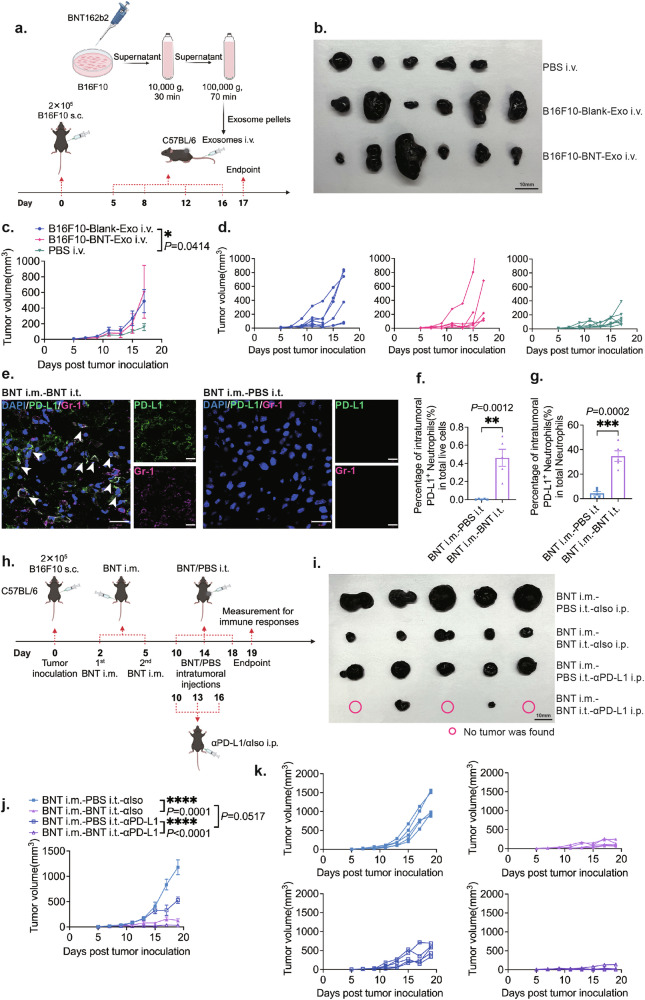
The upregulation of PD-L1 post BNT162b2-based cancer therapy affects the therapeutic efficacy, whereas combinational therapy of BNT162b2 and anti-PD-L1 effectively enhances therapeutic efficacy. **a** Experimental design for investigating the therapeutic efficacy of B16F10-derived exosomes and BNT162b2-transfected B16F10-derived exosomes (*n* = 5 in the PBS i.v. group, *n* = 6 in exosome treatment groups). **b** Photo of tumors from the treatment and control groups at the endpoint of the experiment described in **a**. **c** Tumor growth curves of the BNT162b2-transfected B16F10-derived exosomes intravenous treatment group vs the B16F10-derived exosomes intravenous treatment group vs intravenous PBS control group for the experiment described in **a**. **d** Tumor growth curves of the BNT162b2-transfected B16F10-derived exosomes treatment group, B16F10-derived exosomes treatment group, and PBS control group for the experiment described in **a**. **e** Representative immunofluorescent staining images of tumor-infiltrating PD-L1^+^Gr-1^+^ neutrophils. **f** Quantitative analysis of the percentage of tumor-infiltrating PD-L1^+^Gr-1^+^ neutrophils in total live cells by flow cytometry (*n* = 5 per group). **g** Quantitative analysis of the percentage of tumor-infiltrating PD-L1^+^Gr-1^+^ neutrophils in total neutrophils by flow cytometry (*n* = 5 per group). **h** Experimental design of the combinational therapy of BNT162b2 and anti-PD-L1 (*n* = 5 per group). **i** Photo of tumors from the mice in the BNT162b2 treatment group, BNT162b2 + anti-PD-L1 combinational treatment group, and control groups at the endpoint of the experiment described in **h**. **j** Tumor growth curves of treatment groups vs control groups for the experiment described in **h**. **k** Tumor growth curves of the intratumoral PBS control group, intratumoral BNT162b2 treatment group, intraperitoneal anti-PD-L1 treatment group, and intratumoral BNT162b2 + intraperitoneal anti-PD-L1 combinational treatment group for the experiment described in **h**. BNT i.m.-PBS i.t.-αIso i.p.: the mice with BNT162b2 intramuscular injections, PBS intratumoral injections, and isotype antibody (αIso) intraperitoneal injections. BNT i.m.-BNT i.t.-αIso i.p.: the mice with BNT162b2 intramuscular injections, BNT162b2 intratumoral injections, and isotype antibody (αIso) intraperitoneal injections. BNT i.m.-PBS i.t.-αPD-L1 i.p.: the mice with BNT162b2 intramuscular injections, PBS intratumoral injections, and anti-PD-L1 antibody intraperitoneal injections. BNT i.m.-BNT i.t.-αPD-L1 i.p.: the mice with BNT162b2 intramuscular injections, BNT162b2 intratumoral injections, and anti-PD-L1 antibody intraperitoneal injections. The time points of different administrations were shown in **h**. Scale bars, 20 μm in **e**.

Although tumor-derived exosomes can prime tumor-specific immune responses, many studies showed that tumor-derived exosomes also play a role in facilitating tumor growth^49–51^. Our results on in vivo cancer therapeutic and prophylactic efficacies in mouse models indicated that the intravenous administration of exosomes derived from both B16F10 tumor cells and BNT162b2-transfected B16F10 tumor cells promoted tumor growth (Fig. 5a–d; Supplementary Fig. S7a–d). In addition, PD-L1 has been reported to be present on the surface of tumor-derived exosomes^52^, and these PD-L1-carrying exosomes were shown to contribute to immunosuppression^52,53^. Studies have shown that blockade of the exosome-related PD-1/PD-L1 pathway can induce effective anti-tumor immunity^52,54^. Our transcriptome analysis also indicated a correlation between CD274 (PD-L1) expression and neutrophil or macrophage activation (Fig. 3g). On the other hand, our results demonstrated that after intratumoral injections of BNT162b2, the previously “cold” TME in B16F10 melanoma was transformed into a “hot” TME. This transformation was accompanied by a substantial increase in TILs within the tumor, creating a favorable environment for effective immune checkpoint inhibitors (ICIs)-based therapy to unleash pre-existing anti-tumor immunity^30^. These clues led us to hypothesize that the therapeutic efficacy of BNT162b2-based cancer therapy can be further enhanced by combining with anti-PD-L1 therapy.

We first validated that the exosomes isolated from the B16F10 melanoma cell line carried PD-L1 (Fig. 4m). Since BNT162b2 induces secretion of more exosomes (Fig. 4j, o, p), BNT162b2-based cancer therapy has the potential to induce the release of more PD-L1-carrying exosomes. Immunofluorescent staining of tumor sections also showed that the percentage of PD-L1^+^ cells increased in the tumor after the BNT162b2-based cancer therapy, compared with the control group (Supplementary Fig. S7e, f). The flow cytometry results showed that the expression of PD-L1 on CD45^–^ tumor cells was low, and it did not significantly increase after the BNT162b2-based cancer therapy (Supplementary Fig. S7g). We further analyzed PD-L1 expression on tumor-infiltrating immune cells in tumors by flow cytometry, which revealed a significantly increased CD45^+^ PD-L1^+^ population post BNT162b2-based cancer therapy (Supplementary Fig. S7g). RNA-seq results showed that the expression of PD-L1 was highly correlated with the activation of neutrophils and macrophages (Fig. 3g). Thus, we further investigated the expression of PD-L1 on neutrophils and macrophages by flow cytometry after the intratumoral BNT162b2 treatments. The results showed that after the BNT162b2 treatments, there was not only a significantly increased percentage of intratumoral PD-L1^+^ neutrophils in total live cells in the tumor (Fig. 5f), but also a significantly increased percentage of intratumoral PD-L1^+^ neutrophils in total neutrophils in the tumor (Fig. 5g) when compared to the PBS control group. The increased PD-L1 expression on intratumoral neutrophils was validated by immunofluorescent staining, which also clearly showed an increase in PD-L1^+^ neutrophils after the intratumoral BNT162b2 treatments (Fig. 5e). Further analysis of the CD45^+^PD-L1^+^ population demonstrated a large percentage of macrophages in both the BNT162b2 treatment and control groups (Supplementary Fig. S7h, i). Interestingly, the Gr1^+^ neutrophil population greatly increased post BNT162b2-based cancer therapy and was the dominant population in the CD45^+^PD-L1^+^ cells in the tumor (Supplementary Fig. S7h, i). To study whether PD-L1 expression limits the immunotherapeutic efficacy of BNT162b2, tumor-bearing mice were administered with intratumoral BNT162b2 injections, together with systemic administrations of anti-PD-L1 therapy. (Fig. 5h). Anti-PD-L1 therapy alone demonstrated some inhibition of tumor growth compared to the control group; however, its effect was less significant than that of BNT162b2-based cancer therapy alone (Fig. 5i–k). Importantly, the combinational therapy of BNT162b2 and anti-PD-L1 significantly enhanced the overall therapeutic efficacy compared to either BNT162b2 or anti-PD-L1 therapy alone. As shown in Fig. 5i, B16F10 tumors were completely eliminated in three out of five treated mice, clearly indicating that anti-PD-L1 antibody treatment improved the overall therapeutic efficacy of BNT162b2-based cancer therapy in the melanoma model. Furthermore, we also found some therapeutic efficacy of BNT162b2-based cancer therapy in the advanced melanoma model, and this therapeutic efficacy can be further enhanced by combining anti-PD-L1 therapy as well (Supplementary Fig. S8a–c).

### Safety of BNT162b2-based cancer therapy and combinational therapy of BNT162b2 and anti-PD-L1

Although BNT162b2 has shown strong potential for broad-spectrum cancer therapy, the safety of this therapeutic strategy is also important due to the systemic activation of immune responses during BNT162b2-based cancer therapy. Thus, we assessed the safety and systemic toxicity of BNT162b2-based cancer therapy in mice.

**Fig. 6:**
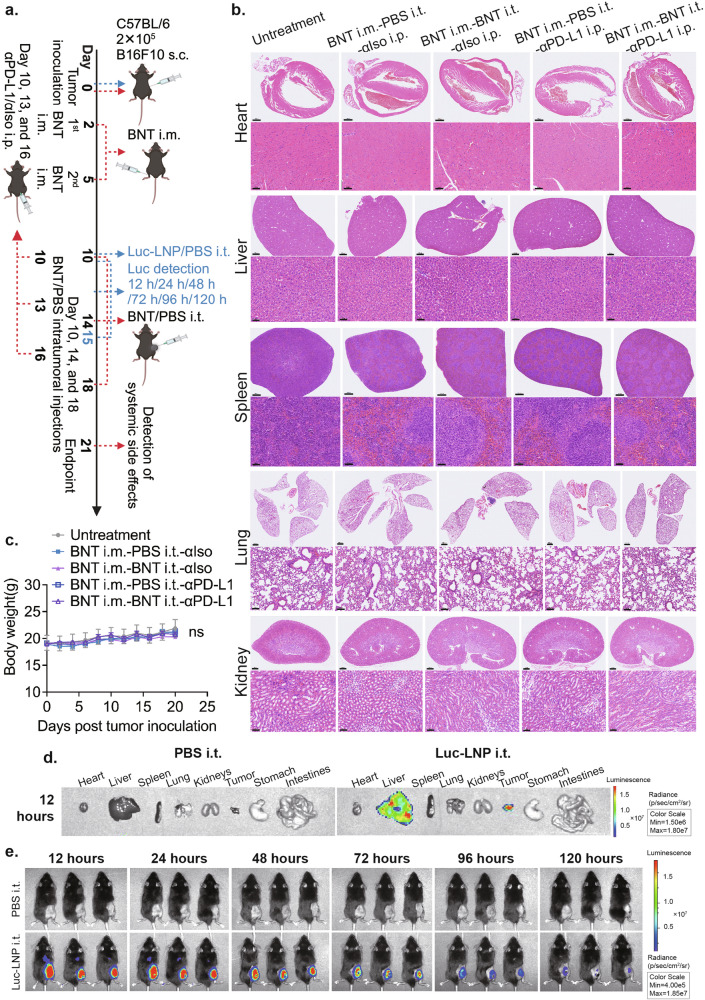
Safety of the BNT162b2-based cancer therapy and the combinational therapy of BNT162b2 and anti-PD-L1. **a** Experimental design for investigating the safety of cancer therapeutic strategies in this study. **b** H&E staining of major organs post different therapeutic strategies. **c** Body weight changes of treatment and control groups during the experimental period. **d** Luciferase signals of major organs after a single-dose Luc mRNA-LNP intratumoral injection. **e** Whole body Luciferase signals after a single-dose Luc mRNA-LNP intratumoral injection. BNT i.m.-PBS i.t.-αIso i.p.: the mice with BNT162b2 intramuscular injections, PBS intratumoral injections, and isotype antibody (αIso) intraperitoneal injections. BNT i.m.-BNT i.t.-αIso i.p.: the mice with BNT162b2 intramuscular injections, BNT162b2 intratumoral injections, and isotype antibody (αIso) intraperitoneal injections. BNT i.m.-PBS i.t.-αPD-L1 i.p.: the mice with BNT162b2 intramuscular injections, PBS intratumoral injections, and anti-PD-L1 antibody intraperitoneal injections. BNT i.m.-BNT i.t.-αPD-L1 i.p.: the mice with BNT162b2 intramuscular injections, BNT162b2 intratumoral injections, and anti-PD-L1 antibody intraperitoneal injections. The time points of different administrations were shown in **a**. In **b**, the scale bars represent the following: 700 μm and 50 μm in the first and second lines of the H&E staining of the heart, respectively; 800 μm and 50 μm in the first and second lines of the H&E staining of the liver, respectively; 600 μm and 50 μm in the first and second lines of the H&E staining of the spleen, respectively; 800 μm and 100 μm in the first and second lines of the H&E staining of the lung, respectively; and 600 μm and 50 μm in the first and second lines of the H&E staining of the kidney, respectively.

Firstly, we conducted a histological study at the endpoint of BNT162b2-based cancer therapy and combinational therapy (Fig. 6a). The haematoxylin & eosin (H&E) staining results of major organs (heart, liver, spleen, lung, kidney) from mice in different treatment groups showed no apparent systemic tissue damage or any sign of inflammatory-related toxicity (Fig. 6b). Additionally, there was no significant difference in body weight changes between the treatment groups and the control groups during the BNT162b2-based cancer therapy period (Fig. 6c). Taken together, these data suggest that both BNT162b2-based cancer therapy and the combinational therapy of BNT162b2 and anti-PD-L1 have no apparent adverse effects during the treatment period.

For a detailed analysis of the transfection efficiency and the distribution of BNT162b2 post-intratumoral injections, we synthesized Luciferase mRNA and encapsulated it in LNP, which has the same design as the LNP used in BNT162b2. Twelve hours after a single dose of intratumoral injection, we found a high-level expression of Luciferase in the tumor. Additionally, we detected an intermediate expression level of Luciferase in the liver and a very low expression level in the spleen (Fig. 6d), indicating that after intratumoral injection, some systemic leakage did occur, but no systemic toxicity was noticed. One possible reason is that the Luciferase protein in the liver and spleen was cleared away quickly after 48 h, while the protein expression in the tumor was maintained for more than five days (Fig. 6e). Additionally, the killing of normal cells in the liver and spleen does not induce further immune reactions. These results indicate that this mRNA vaccine design has great potential in intratumoral drug delivery, and suggests the potential of BNT162b2 in this cancer therapeutic strategy.

### Other pathogen-based mRNA vaccines for cancer immunotherapy

We have demonstrated the cancer therapeutic potential of COVID-19 mRNA vaccine BNT162b2. To determine if this cancer therapeutic approach is a general strategy and extend its potential benefits to more patients, we tested this approach using mRNA vaccines encoding other pathogen antigens.

**Fig. 7:**
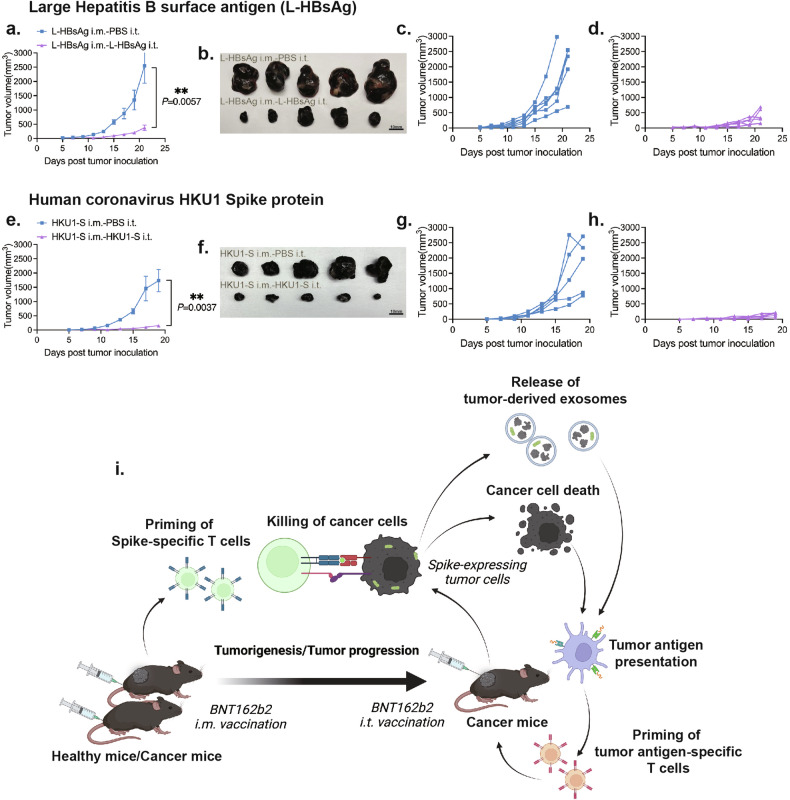
The therapeutic efficacy of other potential pathogen antigen-based mRNA vaccine for cancer therapy and the summary diagram of BNT162b2-based cancer therapy. **a**, **e** Tumor growth curves of the intratumoral L-HBsAg mRNA vaccine (**a**) or HKU1-S mRNA vaccine (**e**) treatment group vs the intratumoral PBS control group. The treatment protocol is the same as the BNT162b2 one. **b, f** Photos of tumors from the mice in the treatment and control groups at the endpoints. **c**, **d**, **g**, **h** Tumor growth curves of intratumoral PBS control group, intratumoral L-HBsAg mRNA vaccine treatment group, and intratumoral HKU1-S mRNA vaccine treatment group. **i** Schematic diagram of the design and mechanism of the BNT162b2-based cancer therapy. In this broad-spectrum cancer therapy design, the memory immunity against spike protein is first established by intramuscular administrations of COVID-19 vaccine, which can be rapidly reactivated to target the tumor cells expressing spike protein due to BNT162b2 COVID-19 mRNA intratumoral injections. This effective BNT162b2-based cancer therapy triggered a potent antigen spreading via the dead cancer cells and tumor-derived exosomes. These tumor antigens containing dead cells or exosomes provoked extensive tumor antigen-specific immune responses (antigen spreading), leading to tumor-specific effective cancer immunotherapy without the need of individualized cancer vaccine. L-HBsAg i.m.-PBS i.t.: the mice with L-HBsAg mRNA vaccine intramuscular injections and PBS intratumoral injections. L-HBsAg i.m.-L-HBsAg i.t.: the mice with L-HBsAg mRNA vaccine intramuscular and intratumoral injections. HKU1-S i.m.-PBS i.t.: the mice with HKU1-S mRNA intramuscular injections and PBS intratumoral injections. HKU1-S i.m.- HKU1-S i.t.: the mice with HKU1-S mRNA vaccine intramuscular and intratumoral injections.

Considering that a large percentage of people have acquired memory immunities against HBV and HCoVs through vaccination or infection^55,56^, we examined the therapeutic efficacy of mRNA vaccines encoding the L-HBsAg of HBV and the HKU1-S of human coronavirus HKU1 in cancer therapy. Our data indicate that both the mRNA vaccine encoding the L-HBsAg of HBV and the mRNA vaccine encoding the HKU1-S of human coronavirus HKU1 can significantly inhibit tumor growth using our cancer therapeutic strategy in the melanoma model (Fig. 7a–h). These results suggest that different therapeutic options could be tailored based on patients’ vaccination history and the memory immunities they still possess.

## Discussion

Immunotherapeutic approaches have seen dramatic clinical success in fighting various types of cancers and in prolonging patients’ lives. However, not all cancer patients benefit from current immunotherapies, partly because of immune escape development due to tumor antigen losses, or antigen-presenting capacity reduction. In addition, an immunosuppressive TME, such as increased intratumoral regulatory immune cells and the increased expression of immune checkpoint proteins, leads to suppression of anti-tumor immune responses. Hence, an enhanced anti-tumor immunotherapy strategy should not only focus on providing a substantial number of active tumor-killing T cells, but also aim to increase tumor antigenicity by presenting more recognizable antigens to effectively activate the tumor immunity cycle and reverse the suppressive TME in favor of anti-tumor immune responses.

This study showcased a potential broad-spectrum cancer therapeutic strategy that mobilizes the acquired memory immunity against infectious diseases and redirects these potent immune responses toward the tumor cells that have been tagged with the same pathogen antigens using mRNA-lipid nanoparticles. Our findings demonstrated that the intratumoral injections of the BNT162b2 vaccine exhibited excellent therapeutic efficacy in treating primary tumor across all tested cancer models in mice that had been immunized with the same vaccine previously. Additionally, the vaccine treatment effectively inhibited tumor lung metastasis in 4T1 breast model. Interestingly, we found that BNT162b2 had a similar anti-cancer effect in the μMT mouse model in comparison to that in WT mice, however, it had no obvious therapeutic effects in Rag1^*−/−*^ mice, indicating that the BNT162b2-based cancer therapy was predominantly T cell-dependent. Further mechanistic studies showed that the BNT162b2-based cancer therapy could effectively recruit immune cells into the tumors, reverse the immunosuppressive TME and activate the intratumoral and systemic anti-tumor immunity to suppress tumor growth. Specifically, our results showed that BNT162b2-based cancer therapy led to increased intratumoral levels of T cells, B cells, NK cells, macrophages, APCs, and neutrophils. In addition, MHC expression was also increased in the tumor, which improved the efficiency of antigen presentation in the tumor. Furthermore, the BNT162b2 intratumoral treatment resulted in a shift of the phenotypes of intratumoral macrophages from M2 toward M1. The cytokine profiling in the tumor after BNT162b2-based cancer therapy showed a trend that was proinflammatory and anti-tumor. The intratumoral BNT162b2 treatments not only reactivated and redirected the anti-spike protein T cell response to target tumor cells expressing the spike protein, but also stimulated T cell responses against tumor antigens through antigen spreading. Upon further investigation of the antigen spreading process, we discovered that BNT162b2 treatment-induced antigen spreading was associated with increased tumor cell death, elevated expression levels of antigen presentation-related HSPs, and an increased release of tumor cell-derived exosomes.

Although the BNT162b2-based cancer therapy showed excellent tumor growth inhibition in the B16F10 melanoma model, it did not completely eliminate the tumor. Thus, we investigated the changes of TME post BNT162b2-based cancer therapy, anticipating to further improve the outcome of BNT162b2-based cancer therapy by TME regulation. The results showed that BNT162b2-based cancer therapy did not significantly increase the PD-L1 expression level on tumor cells, but greatly increased PD-L1 expression in the CD45^+^ leukocyte population. Furthermore, the flow cytometry analysis revealed increased numbers of PD-L1^+^ CD11b^+^ F4/80^–^Gr1^+^ neutrophils post BNT162b2-based cancer therapy. In fact, PD-L1^+^ CD11b^+^ F4/80^–^Gr1^+^ neutrophils and PD-L1^+^ CD11b^+^ F4/80^+^ macrophages accounted for the majority of PD-L1^+^ leukocytes post BNT162b2-based cancer therapy. Moreover, there was an increased release of tumor-derived PD-L1-containing exosomes. Thus, the elevated PD-L1^+^ tumor-derived exosomes, intratumoral PD-L1^+^ neutrophils, intratumoral PD-L1^+^ macrophages, and PD-L1^+^ tumor cells all have the potential to attenuate the anti-tumor responses elicited by BNT162b2-based cancer therapy. Taking into account the significant increase in TILs after BNT162b2 intratumoral injections in the B16F10 “cold tumor” model, the TME following BNT162b2-based cancer therapy exhibited a “hot tumor” phenotype. Consequently, there is potential for ICI-based therapy to enhance the therapeutic efficacy of BNT162b2-based cancer treatment by reinvigorating the intratumoral immune response^30^. Indeed, we found that the combinational therapy of anti-PD-L1 and BNT162b2 significantly enhanced therapeutic efficacy, which led to complete elimination of the tumor in some cases. Our data also indicated an effective tumor inhibition in the advanced melanoma model, especially when we combined anti-PD-L1 therapy with BNT162b2-based cancer therapy. Interestingly, although the B16F10 tumor model is resistant to anti-PD-L1 monotherapy, our data suggested that the BNT162b2-based cancer therapy restored the therapeutic efficacy of anti-PD-L1 therapy by converting the TME into a “hot tumor” phenotype.

Despite the profound local inflammation reaction and excellent antitumor therapeutic efficacy, we observed extremely low systemic toxicity induced by the BNT162b2-based cancer therapy and the combinational therapy of BNT162b2 and anti-PD-L1. Our data also revealed that the expression of exogenous protein introduced by mRNA vaccine persisted for a long-time period (more than 5 days) in tumor tissue, but only for a short duration (less than 48 h) in some major organs, such as liver and spleen. This observation, along with the safety record based on the clinical data from the use of BNT162b2, leads us to believe that BNT162b2-based cancer immunotherapy can achieve significant therapeutic efficacy without the risk of serious systemic toxicity.

Compared to the conventional cancer vaccine design, the strategy of repurposing BNT162b2 as a simple, ready-to-use, and broad-spectrum cancer therapy, has clear advantages over the expensive and time-consuming individualized approach. More importantly, we emphasize that the intratumoral injection of BNT162b2 effectively acts as a precision-guiding agent that mobilizes existing immunity. The speed and magnitude of this response are significantly more profound than those of a comparable vaccine that serves as a priming agent for the host. In this regard, the establishment of host memory immunity against the antigen used to tag the tumor is essential. This is evidenced by our control experiments, where direct intratumoral injection of mRNA lipid nanoparticles encoding foreign antigens without pre-existing memory immunity against the same antigen resulted in a much less therapeutic effect.

We would also like to stress that anti-SARS-CoV-2 spike protein immunity already exists in the majority of the global population, either through full or partial vaccinations with different forms of COVID-19 vaccines or through SARS-CoV-2 virus infections. One practical advantage of the BNT162b2-based cancer therapeutic strategy is that BNT162b2 and other mRNA, recombinant protein, or inactivated virus-based COVID-19 vaccines have already been approved for clinical use and have demonstrated to be effective and safe. Consequently, the BNT162b2 vaccine could be quickly approved for clinical trials as a cancer therapeutic agent.

In addition to the SARS-CoV-2 spike protein, we anticipate that other strong antigens derived from human or animal pathogens may also serve similar anti-tumor roles as demonstrated by the SARS-CoV-2 spike protein in this study. Our data suggest that the L-HBsAg mRNA vaccine and HCoVs HKU1-S mRNA vaccine also exhibit significant therapeutic efficacy for cancer treatment. This not only validates this strategy for cancer therapy but also provides additional treatment options for cancer patients based on their vaccination history and the current level of immunity against specific pathogens. During the revision of this work, we noticed that Boehm et al. posted a manuscript on bioRxiv^57^, demonstrating that the intratumoral administration of SpikeVax (mRNA-1273), a COVID-19 vaccine by Moderna, delays melanoma growth in mice. Our study, however, shows that, in addition to direct intratumoral administration, establishing memory immunity against SARS-CoV-2 before intratumoral injection can lead to more rapid and potent anti-tumor immune responses.

In summary, as illustrated in Fig. 7i, our BNT162b2-based cancer therapeutic strategy can efficiently and swiftly mobilize anti-spike immune memory to target tumor cells expressing the spike protein, induced by intratumoral BNT162b2 injections. The effective elimination of spike protein-tagged cancer cells initiates a cascade of tumor-specific immune responses through antigen spreading in a reprogrammed TME, and eventually leads to a successful cancer immunity cycle. Furthermore, the efficacy of this therapeutic approach can be augmented by combining with anti-PD-L1 therapy. This study highlights the potential of using the COVID-19 mRNA vaccine as a potent, broad-spectrum cancer treatment agent with strong therapeutic efficacy. It also suggests the application potential of other pathogen antigen-based mRNA vaccines for cancer immunotherapy.

## Materials and methods

### Cancer cell lines and vaccine

Mouse MB49 bladder cancer, mouse 4T1 breast cancer, mouse CT26 colon cancer, and mouse B16F10 melanoma cancer cell lines were purchased from ATCC. The 4T1 and B16F10 cell lines were cultured in RPMI Medium 1640 with 10% fetal bovine serum (FBS). The MB49 and CT26 cell lines were cultured in Dulbecco’s Modified Eagle Medium (DMEM) with 10% FBS. All cells were cultured at 37 °C in 5% CO_2_. Leftover Pfizer/BioNTech BNT162b2 (Comirnaty) mRNA vaccines, and SinoVac COVID-19 inactivated vaccines (CoronaVac) were obtained in their original vials from the Department of Health, HKSAR. The BNT162b2 mRNA vaccine was stored at −80 °C until use, the SinoVac inactivated vaccine was stored at 4 °C until use. Luciferase mRNA vaccine, HBV mRNA vaccine, and HCoV HKU1 mRNA vaccine were provided by Scindy Pharmaceutical.

### Mice, tumor challenge, and treatment

The animal protocol was approved by the Committee on the Use of Live Animals in Teaching and Research (CULATR), The University of Hong Kong. The C57BL/6 and BALB/c mice (6–8 weeks) were purchased from The Centre for Comparative Medicine Research (CCMR) in the LSK Faculty of Medicine, The University of Hong Kong. The μMT and Rag1^*−/−*^ mice were purchased from The Jackson Laboratory.

Treatment scheme 1: Mice were randomly assigned to two groups. On Days –42 and –21, all mice received a dose of an intramuscular injection of 50 µL diluted BNT162b2 vaccine in saline containing 5 µg mRNA. On Day 0, the vaccinated C57BL/6 mice were subcutaneously inoculated with 3 × 10^5^ B16F10 tumor cells. On Days 5, 10, and 17, each mouse in the treatment group received an intratumoral injection of 50 µL diluted BNT162b2 vaccine, whereas the control group received intratumoral injection of an equal amount of PBS.

Treatment scheme 2: mice were randomly assigned to two groups. On Day 0, 2 × 10^5^ B16F10 tumor cells or 1 × 10^5^ MB49 tumor cells were implanted subcutaneously into C57BL/6 mice, 2 × 10^5^ CT26 tumor cells were implanted subcutaneously into BALB/c mice, and 2 × 10^5^ 4T1 tumor cells were implanted into the mammary fat pad of BALB/c mice. On Days 2 and 5, all mice received intramuscular injections of 50 µL diluted BNT162b2 vaccine in saline containing 5 µg mRNA, or 50 µL SinoVac, or 10 µg RBD protein, or 50 µg RBD protein. On Days 10, 15, and 20 or on Days 10, 14, and 18, mice in the treatment group received intratumoral injections of 50 µL diluted BNT162b2 containing 5 µg mRNA, or 50 µL SinoVac, or 10 µg RBD protein, or 50 µg RBD protein, whereas the mice in the control group received intratumoral injections of an equal amount of PBS.

In combinational therapy: the experiment was performed as treatment scheme 2. 250 µg of anti-PD-L1 antibodies (BioXCell, BE0101) or isotype control antibodies (BioXCell, BE0090) was injected intraperitoneally right after the first dose of BNT162b2 or PBS intratumoral injections, and the anti-PD-L1 treatment was repeated once every three days throughout the duration of BNT162b2 or PBS intratumoral treatments.

### Immunofluorescent staining

Tumor specimens obtained from sacrificed mice were embedded in the Tissue-Tek O.C.T Compound (SAKURA, 4583) and frozen. Next, 8-μm sections were prepared and blocked with 5% BSA, followed by incubation with primary antibodies shown in Supplementary Table S1 in 1% BSA, at 4 °C overnight. Slides were washed three times with 1× TBST, 5 min each time, and then the slides were stained with secondary antibodies shown in Supplementary Table S1 at room temperature for 1 h. Slides were washed three times with 1× TBST, 5 min each time, and then stained with DAPI at room temperature for 20 min. Slides were washed three times with 1× TBST, 5 min each time, and images were acquired using a Carl Zeiss LSM880 confocal microscope.

To stain for intracellular spike protein and CD63, tumor cryosections were incubated with PBS containing 0.1% Triton X-100 for 10 min, washed three times with 1× TBST, 5 min each time. It was followed by 5% BSA blocking and antibody incubations as described above.

### Flow cytometry

Cell suspensions were prepared from tumor tissues by using Collagenase I and IV (1 mg/mL each, Sigma-Aldrich, SCR103 and C5138) and a gentle MACS Dissociator (Miltenyi Biotec, 130-093-235). Cells were stained with antibodies shown in Supplementary Table S2. Flow cytometry and data analysis were performed on an Agilent NovoCyte Quanteon analyzer. The gating strategy is shown in Supplementary Fig. S2a.

To stain intracellular HSPs (Calreticulin, HSP70), IFN-γ, and CD63, cells were treated with BD Cytofix/Cytoperm Fixation/Permeabilization Kit (BD, 554714). Antibodies shown in Supplementary Table S3 were used. For the unlabeled anti-Calreticulin primary antibody, Alexa Fluor 488-labeled goat anti-mouse IgG (H + L) cross-absorbed secondary antibody shown in Supplementary Table S3 was used.

### Cytokine profiling

Blood samples and tumor tissues were collected from mice in the BNT162b2 treatment group and the control group at the endpoint for cytokine profiling (BioLegend, LEGENDplex MU Th Cytokine Panel (12-plex), 741043 and LEGENDplex Mouse Inflammation Panel (13-plex), 740446).

Serum samples were prepared by centrifugation at 5000*×* *g* for 30 min at room temperature. Tumor cytokine levels were measured in supernatants of tissue homogenates. Tissue homogenates were prepared by using PowerBead Tubes (QIAGEN, 19301) and a BeadBug Benchtop Homogenizer (Benchmark Scientific), and supernatants were obtained after centrifugation at 5000*×* *g* for 20 min at 4 °C.

### RNA-seq and data analysis

Total RNA was extracted from tumor tissues by Trizol and chloroform followed by precipitation in 2-propanol and ethanol. RNA-seq was carried out by Novagene Co., Ltd. Gene expression was quantitated by ‘Salmon’ directly from the bulk transcriptome sequencing data^58^. DESeq2 was used for the differential analysis of gene expression using default parameters^59^. GO analysis was limited to immune-related biology processes (ontologies belonging to GO:0002376) and GO terms were merged according to the similarity between each ontology with the help of clusterProfiler. Immune cell functional enrichment was conducted by xCell using a mouse gene expression profile with the mouse gene symbol transferred to the human gene symbol^60,61^.

### ELISA

96-well ELISA plates (JET BIOFIL, FEP-100-096) were coated with 0.1 μg/mL spike protein or 10 μg/mL tumor membrane proteins (extracted by Pierce Mem-PER Plus Membrane Protein Extraction Kit, Thermo Fisher, 89842) in coating buffer (BioLegend, 421701) and incubated at 4 °C overnight. The plates were blocked by blocking buffer (BIO-RAD, 1706404, the powder was dissolved in 1× TBST) and incubated at room temperature for 2 h. To measure the serum antibody titers against the spike protein, serum samples were serially diluted at 1:150, 1:450, 1:1350, 1:4050, 1:12,150, 1:36,450, 1:109,350, 1:328,050, 1:984,150, or 1:2,952,450 in blocking buffer, and plates were incubated for 2 h at room temperature. To measure serum antibody titers against tumor membrane proteins, serum samples were serially diluted at 1:15, 1:45, 1:135, 1:405, 1:1215, 1:3645, 1:10,935, or 1:32,805 in blocking buffer, and plates were incubated for 2 h at room temperature. After washing three times in 1× TBST, samples were incubated with horseradish peroxidase (HRP)-conjugated goat anti-mouse IgG (1:5000, GE Healthcare) for 1 h at room temperature. After plates were washed five times in 1× TBST, 100 μL of HRP substrate (TMB Chromogen Solution, Thermo Fisher, 002023) was added to each well. After incubation for 15 min, 50 μL of 2 M H_2_SO_4_ solution was added to stop the reaction. The ELISA plates were analyzed by an absorbance microplate reader (Varioskan Flash, Thermo Fisher) at 450 nm wavelength. AUC was calculated and data analysis was performed in GraphPad Prism.

### ELISpot assay

ELISpot assay was performed using the mouse IFN-γ ELISpot PLUS (HRP) Kit (3321-4HST-2, Mabtech). Splenocytes were obtained from sacrificed mice from the treatment and control groups. Splenocytes were stimulated 20–24 h at 37 °C in 5% CO_2_ with one of the following: a peptide pool of spike protein (Mabtech, 3630-1), 27-mer chicken ovalbumin (OVA) peptides containing SIINFEKL (OVA-MHCI: EVSGLEQLESIINFEKLTEWTSSNVME) or ISQAVHAAHAEINEAGR (OVA-MHCII: AESLKISQAVHAAHAEINEAGREVVGS), a pool of MB49 neoantigen peptides (we identified several potential MB49-specific cancer neoantigens with good therapeutic effects, data not shown), B16F10 cell lysate, liquid nitrogen-treated B16F10 (LNT-B16F10), or cell stimulation cocktail positive control (Thermo Fisher, 00-4970-03), respectively. The B16F10 cell lysate was prepared by liquid nitrogen freeze-thaw treatment several times. For freeze-thaw treatment, B16F10 was snap-frozen in liquid nitrogen, then thawed at room temperature. The LNT-B16F10 was prepared by cryopreserving B16F10 cells in FBS + 10% DMSO directly in the liquid nitrogen tank without any slow programmable freezing method. ELISpot plates were imaged by CTL ImmunoSpot ELISpot Analyzer. Data were statistically analyzed with GraphPad Prism by Student’s *t*-test.

### Development of a stable OVA-expressing B16F10 tumor cell line

Lentivirus for expressing chicken OVA was produced in a LentiX cell line (was purchased from ATCC) and collected in the medium twice every 24 h. The collected lentivirus was pooled, concentrated by ultracentrifugation at 126,100*×* *g* for 2 h at 4 °C, lentivirus pellets were resuspended in PBS, and frozen at –80 °C. The B16F10-OVA cell line was developed by infecting with the concentrated lentivirus at an MOI of 1. B16F10-OVA cells that expressed high levels of OVA were selected by cell sorting using flow cytometry (BD FACSAria Fusion).

### Transfection of tumor cell line with BNT162b2

The B16F10-OVA or B16F10 cells (2–3 × 10^6^) in 100 mm cell culture dishes were transfected with BNT162b2 containing 1.0–1.5 μg mRNA in RPMI-1640 supplemented with 10% exosome-depleted FBS (Thermo Fisher, A2720803). The BNT162b2 mRNA transfection was performed every 24 h, and repeated 2–3 times. Each time before transfection, the supernatant post 24 h transfection was collected and stored at 4 °C for exosome isolation, fresh medium was resupplied.

The B16F10 or B16F10-OVA cells in 6 well plates were transfected with BNT162b2 containing 0.8–1.0 μg mRNA each well; the transfection was repeated 2–3 times every 24 h. After 2–3 times transfection, cells were prepared and used for CD63 or HSPs (Calreticulin, HSP70) detections by flow cytometry or immunofluorescent staining.

### Exosome isolation

The pooled medium was centrifuged at 2000*×* *g* for 20 min at 4 °C. The supernatant was filtered through a 0.45-μm filter and cellular debris was removed by centrifugation at 10,000*×* *g* for 30 min at 4 °C. Exosomes were isolated by ultracentrifugation at 100,000*×* *g* for 70 min at 4 °C. The pellet was washed in 60 mL PBS and ultracentrifuged again at 100,000*×* *g* for 70 min at 4 °C. The exosomes were resuspended in PBS and stored at –80 °C.

### Western blotting analysis

Naïve B16F10- or B16F10-OVA-derived exosomes were lysed in Mammalian Protein Extraction Reagent (Thermo Fisher, 78501) with a protease inhibitor cocktail (Roche, 04693132001). The proteins were separated by SDS-PAGE and then transferred to PVDF membranes. After 60 min blocking with 5% Blotting-Grade Blocker (BIO-RAD, 1706404), PVDF membranes were incubated with CD63 polyclonal antibody (Thermo Fisher, PA5-92370), TSG101 monoclonal antibody (Thermo Fisher, MA1-23296), Ovalbumin monoclonal antibody (Thermo Fisher, MA5-41656), SARS Coronavirus spike protein polyclonal antibody (Thermo Fisher, PA1-41165), or InVivoMAb anti-mouse PD-L1 (BioXCell, BE0101) primary antibodies overnight at 4 °C. Then, membranes were incubated with horseradish peroxidase-conjugated anti-rabbit, anti-mouse, or anti-rat IgG secondary antibodies (Thermo Fisher, 31460, 31430, 31470) for 1 h at room temperature. The protein bands were detected by an enhanced chemiluminescence (ECL) detection system (Thermo Fisher, Pierce ECL Western Blotting Substrate, 32106).

### Statistical analysis

Data analysis was performed using GraphPad Prism. Statistical analysis between two groups was performed by unpaired two-tailed Student’s *t*-test or Kolmogorov-Smirnov test. The survival rate data were analyzed by the Log-rank (Mantel-Cox) test. *P* < 0.05 was considered statistically significant. All data are presented as mean values ± SEM.

## Supplementary information

Supplementary Information

## Data Availability

The raw bulk RNA sequencing data reported in this paper have been deposited in the NCBI Sequence Read Archive under BioProject accession number PRJNA1165481.
