# Supplementary material for: The guided fire from within: intratumoral administration of mRNA-based vaccines to mobilize memory immunity and direct immune responses against pathogen to target solid tumors
Source: Cell Discov. 2025 Jan 2;10:127. doi: 10.1038/s41421-024-00743-3 (PMC11693766; doi:10.1038/s41421-024-00743-3)
Supplement: Supplementary file 1 — Supplementary Information [file 41421_2024_743_MOESM1_ESM.pdf]

**Supplementary information for**

# The Guided Fire from Within: Intratumoral Administration of mRNA-Based Vaccines to Mobilize Memory Immunity and Direct Immune Responses against Pathogen to Target Solid Tumors

Renhao Li[1,2]†, Jing-Chu Hu[1]†, Li Rong[1]†, Yige He[1]†, Xiaolei Wang[1,4], Xuansheng Lin[1], Wenjun Li[1], Yangfan Wu[1,2], Chaiyaporn Kuwentrai[1], Canhui Su[1], Thomas Yau[2], Ivan Fan-Ngai Hung[2]*, Xiang Gao[7]*, Jian-Dong Huang[1,3,4,5,6]*

Corresponding author: jdhuang@hku.hk

**The file includes:**

Supplementary Figures S1 to S8

Supplementary Tables S1 to S3

## Supplementary Fig. S1

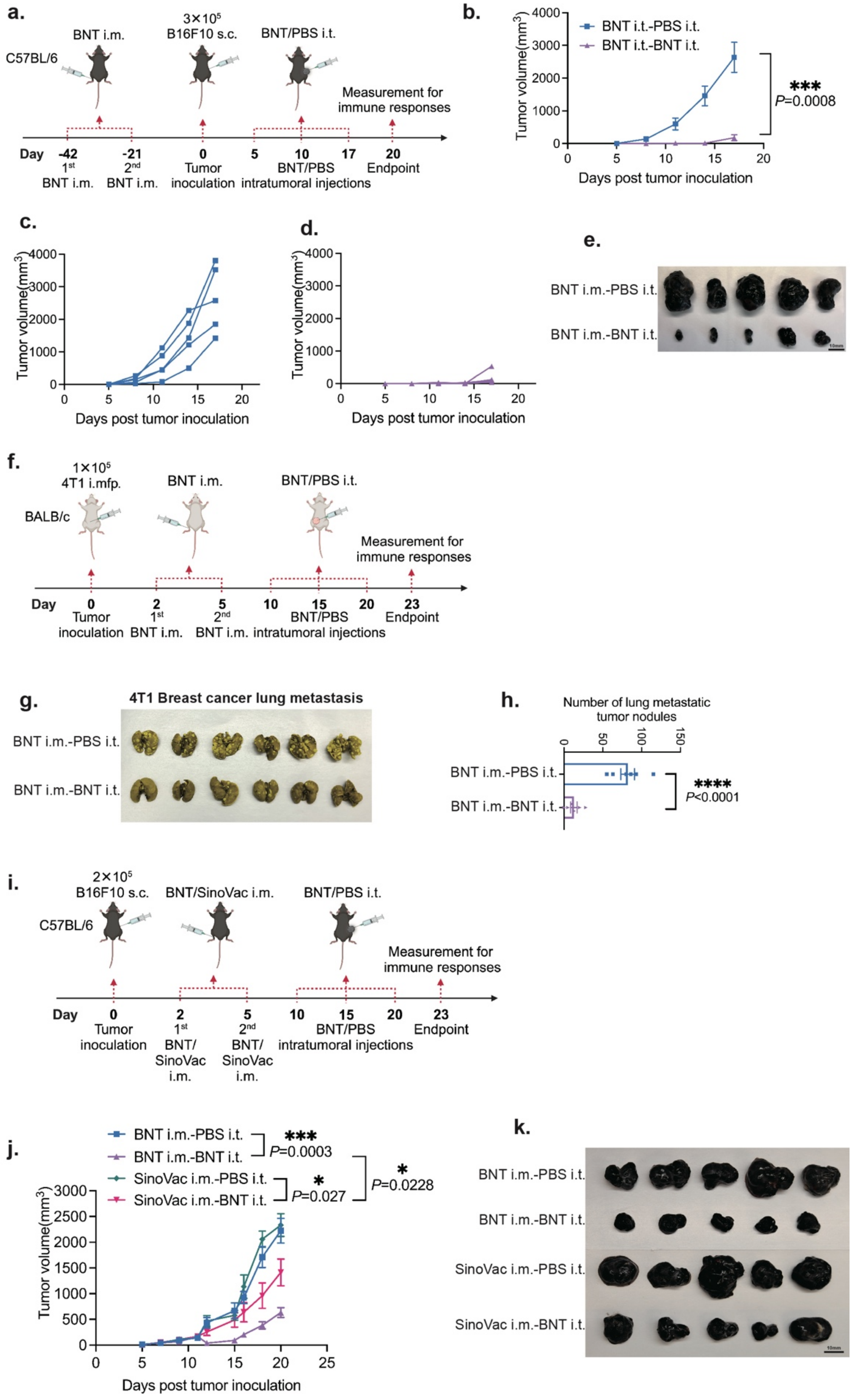

**Supplementary Fig. S1. The therapeutic efficacy of BNT162b2-based cancer therapy.**

**(a)** Experimental design of BNT162b2-based cancer therapy. Intramuscular BNT162b2 vaccinations were carried out before cancer cell implantation (*n* = 5 per group). **(b)** Tumor growth curves of the intratumoral BNT162b2 injections treatment group (BNT i.m.-BNT i.t. group) vs. the intratumoral PBS injections control group (BNT i.m.-PBS i.t. group) for the experiment described in (a). **(c)** Tumor growth curves of the intratumoral PBS injections control group (BNT i.m.-PBS i.t. group). **(d)** Tumor growth curves of the intratumoral BNT162b2 injections treatment group (BNT i.m.-BNT i.t. group). **(e)** Photo of tumors from the treatment (BNT i.m.-BNT i.t. group) and control (BNT i.m.-PBS i.t. group) groups at the endpoint of the experiment described in (a). **(f)** Experimental design of BNT162b2-based cancer therapy in 4T1 breast cancer model. Intramuscular BNT162b2 vaccinations were carried out after cancer cell implantation (*n* = 6 per group). (**g**) Photo of lung metastasis of 4T1 breast cancer in treatment (BNT i.m.-BNT i.t.) and control groups (BNT i.m.-PBS i.t.) at the endpoint of the experiment described in (f). **(h)** Quantification of 4T1 lung metastatic nodules on the lung surface. BNT i.m.-PBS i.t. (control group): the mice with BNT162b2 intramuscular injections and PBS intratumoral injections. BNT i.m.-BNT i.t. (treatment group): the mice with BNT162b2 intramuscular injections and BNT162b2 intratumoral injections. **(i)** Experimental design of BNT162b2-based cancer therapy in mice got anti-spike memory immunity by SinoVac (CoronaVac), COVID-19 inactive virus vaccine, intramuscular injections. **(j)** Tumor growth curves of the mice got intramuscular BNT162b2 injections and intratumoral PBS injections (BNT i.m.-PBS i.t. control group) vs. the mice got intramuscular and intratumoral BNT162b2 injections (BNT i.m.-BNT i.t. treatment group) vs. the mice got intramuscular SinoVac injections and intratumoral PBS injections (SinoVac i.m.-PBS i.t. control group) vs. the mice got

intramuscular and intratumoral SinoVac injections (SinoVac i.m.- SinoVac i.t. treatment group) for the experiment described in (i). **(k)** Photo of tumors from the treatment (BNT i.m.-BNT i.t., and SinoVac i.m.- SinoVac i.t.) and control (BNT i.m.-PBS i.t., and SinoVac i.m.-PBS i.t.) groups at the endpoint of the experiment described in (i).

**Supplementary Fig. S2**

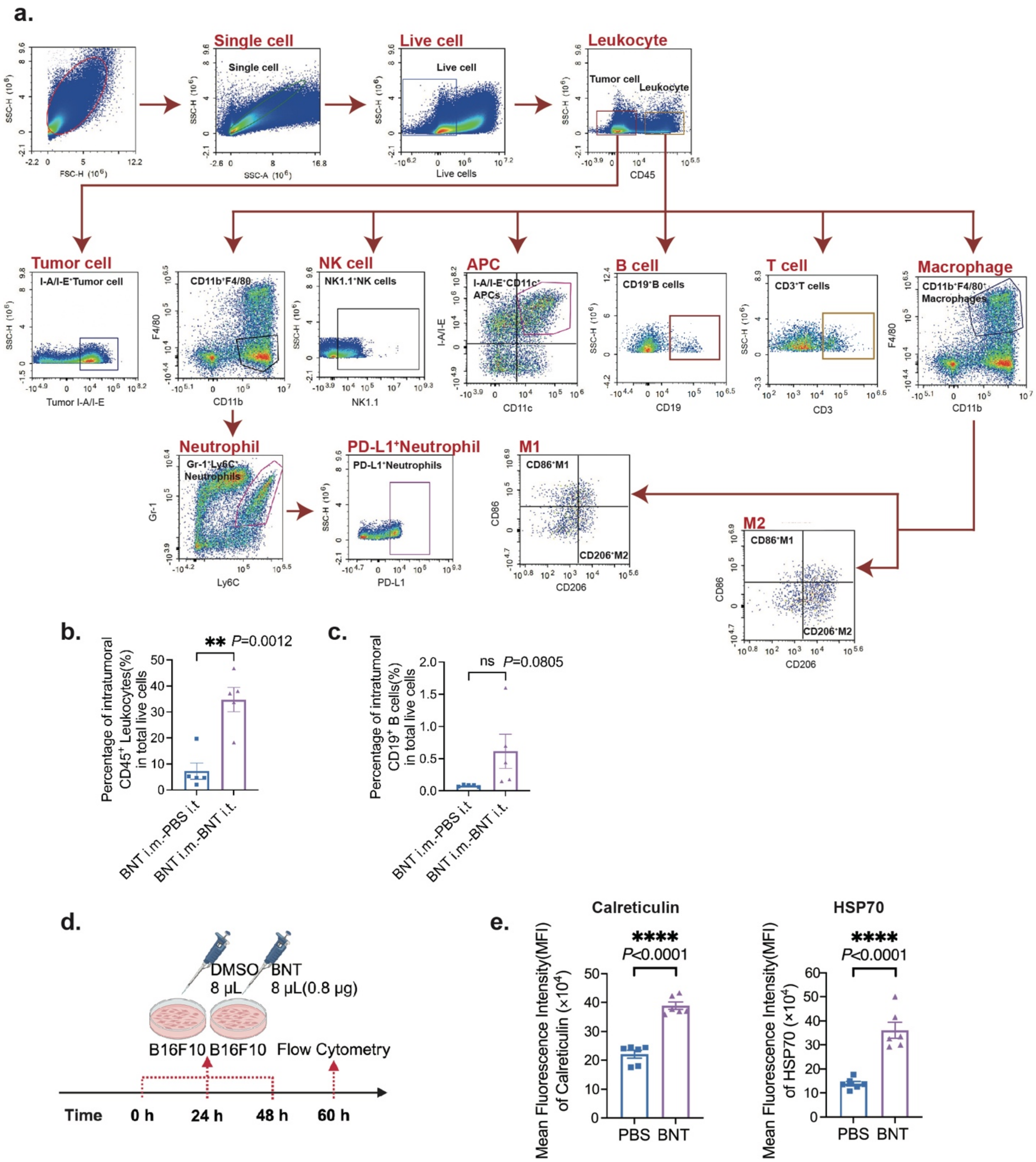

**Supplementary Fig. S2. Flow cytometry to identify the intratumoral immune cell populations.**

**(a)** Gating strategy of the flow cytometry analysis of the intratumoral immune cell populations. **(b)** Quantitative analysis of tumor-infiltrating $CD45^+$ leukocytes by flow cytometry (*n* = 5 per group). **(c)** Quantitative analysis of tumor-infiltrating $CD19^+$ B cells by flow cytometry (*n* = 5 per group). **(d)** Experimental design for investigating the level of heat shock proteins (HSPs) in tumor cells after BNT162b2 treatments. **(e)** The level of HSPs (Calreticulin, HSP70) in tumor cells post PBS treatments or BNT162b2 treatments. For cells with different treatments, the respective mean fluorescence intensity (MFI) of unstained control was subtracted.

**Supplementary Fig. S3**

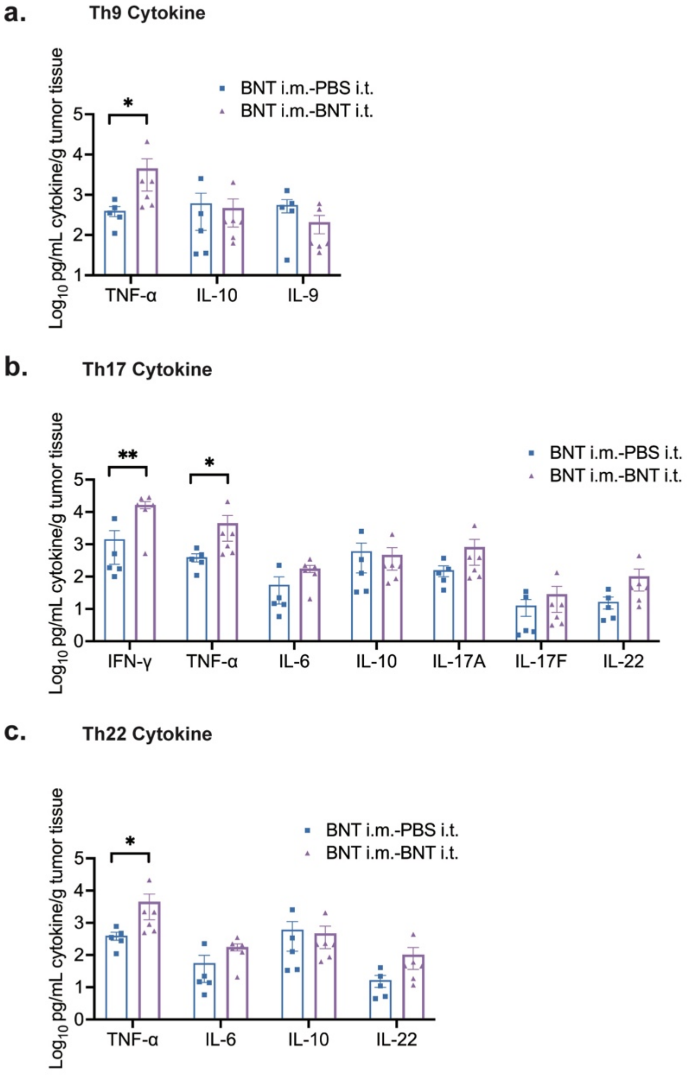


**Supplementary Fig. S3. Analysis of TME by cytokine profiling of the tumor.**

**(a)** Intratumoral Th9-associated cytokine analysis ($n$ = 5 in BNT i.m.-PBS i.t. group, $n$ = 6 in BNT i.m.-BNT i.t. group). **(b)** Intratumoral Th17-associated cytokine analysis ($n$ = 5 in BNT i.m.-PBS i.t. group, $n$ = 6 in BNT i.m.-BNT i.t. group). **(c)** Intratumoral Th22-associated cytokine analysis ($n$ = 5 in BNT i.m.-PBS i.t. group, $n$ = 6 in BNT i.m.-BNT i.t. group). * represents $P < 0.05$; ** represents $P < 0.01$.

**Supplementary Fig. S4**

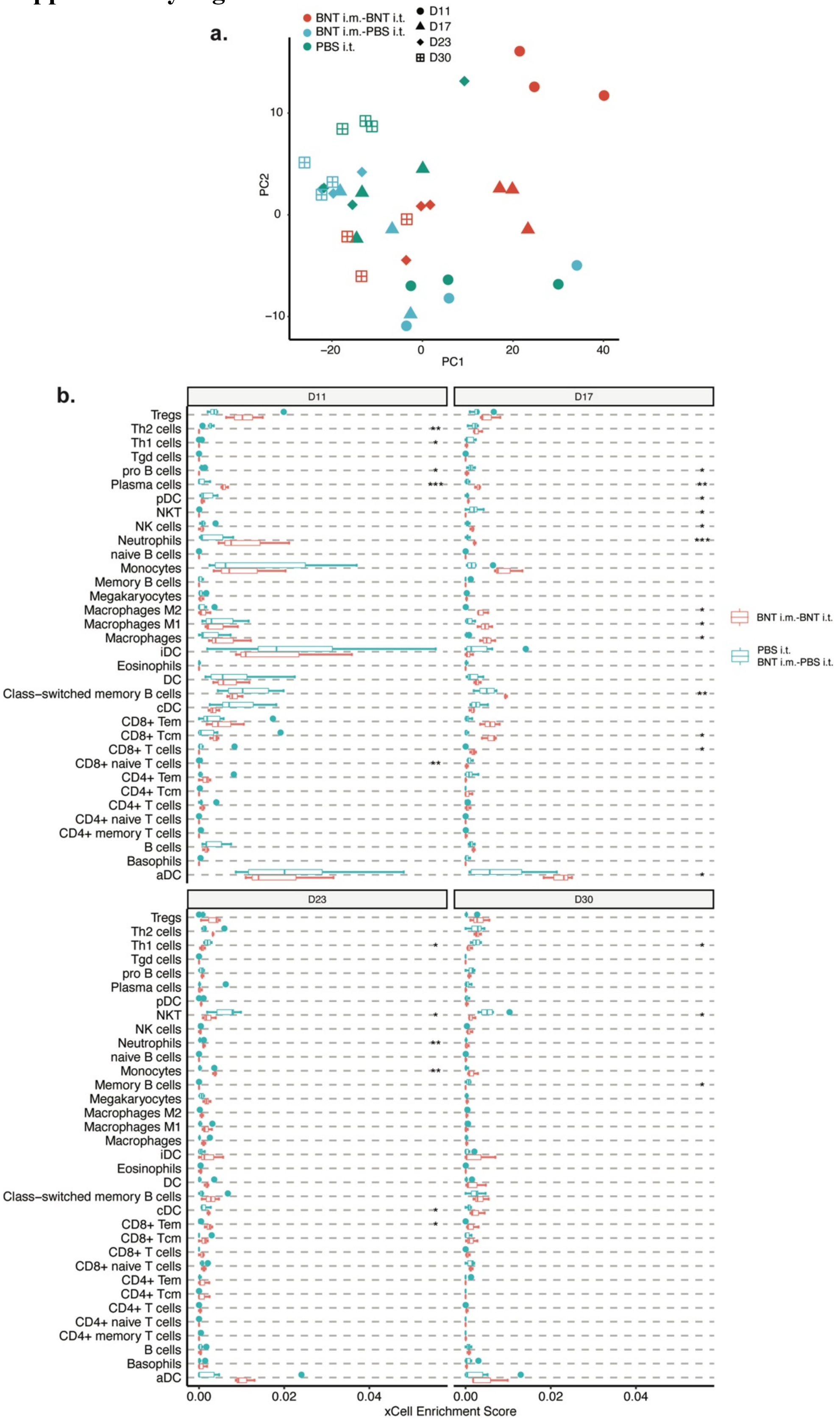

**Supplementary Fig. S4. Bulk RNA-seq data analysis.**

**(a)** Principal component analysis (PCA) of the RNA-seq data (the first two components are shown). **(b)** Inferred activation score of selected immune cell types. t-test was used to analyze statistical differences. * represents $P < 0.05$; ** represents $P < 0.01$; *** represents $P < 0.001$; ns, not statistically significant. $n = 3$ in each timepoint in the BNT i.m.-BNT i.t. group, BNT i.m.-PBS i.t. group, or PBS i.t. group (mice only got PBS intratumoral injections).

**Supplementary Fig. S5**

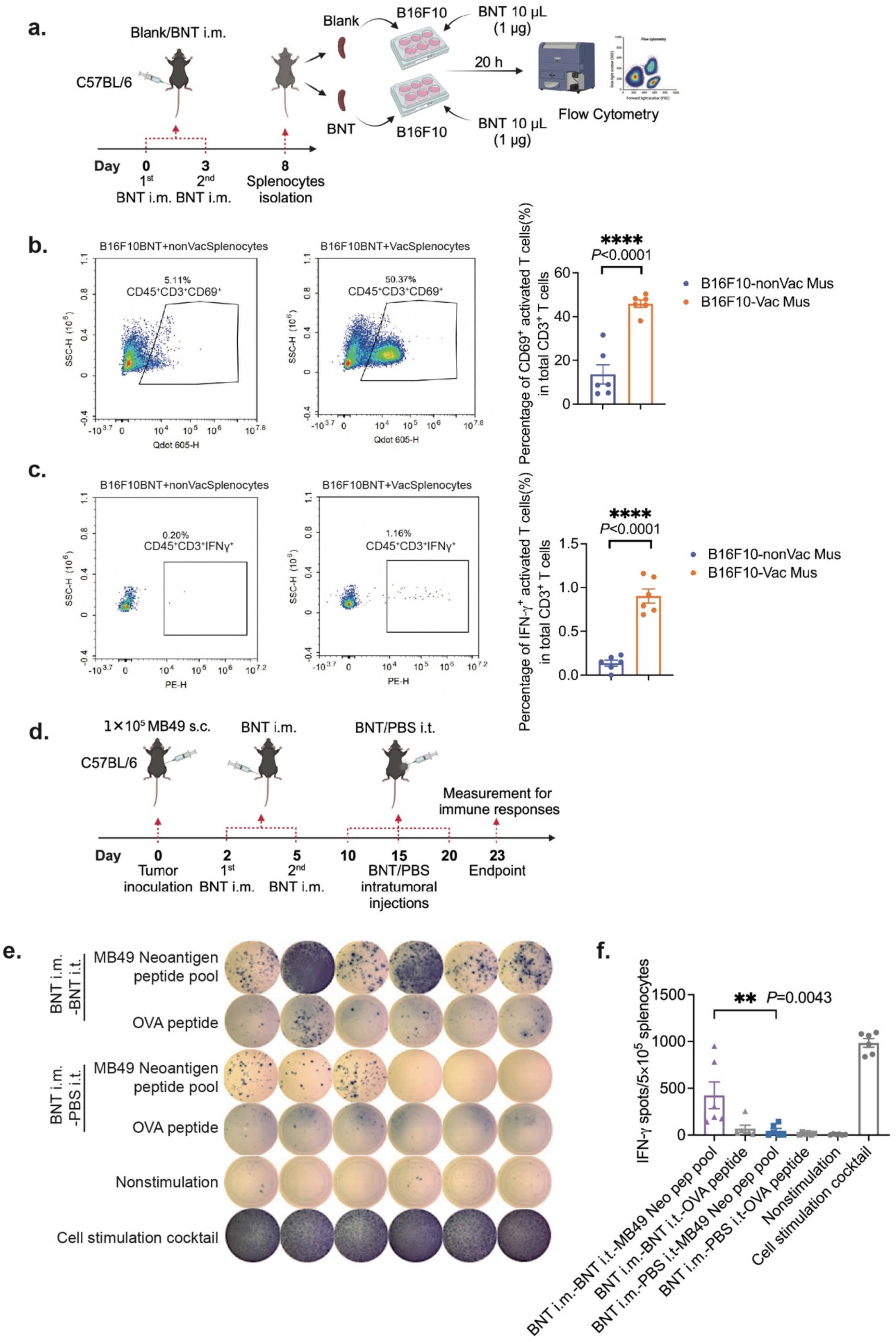

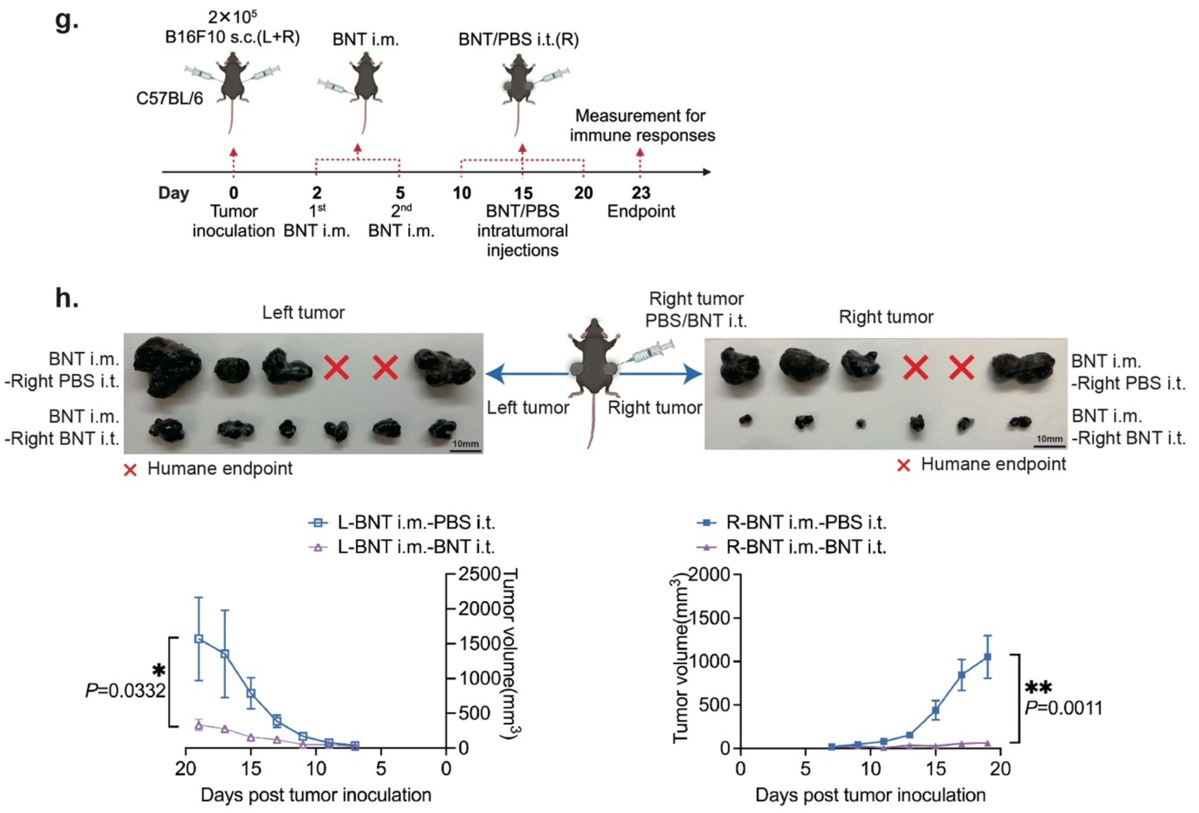


**Supplementary Fig. S5. BNT162b2-based cancer therapy induces rapidly T cell activation and potently systemic tumor antigen spreading.**

**(a)** Experimental design for the validation of the rapid T cell activation post BNT162b2 treatments. B16F10 cells were cultured in 6-well plates. When B16F10 cells are around 80% confluent in 6-well plates, splenocytes were collected from mice who got BNT162b2 intramuscular vaccination or didn't get any vaccination, $2.5\times10^6$ splenocytes were added into each well of plates. At the same time, BNT162b2 was added into 6-well plates. Flow cytometry was performed to detect T cell activation 20 hours post BNT162b2 transfection. **(b)** Comparison of the percentage of $CD69^+$ activated T cells post different treatments as the experimental design described in (a). **(c)** Comparison of the percentage of IFN-$\gamma^+$ activated T cells post different treatments as the experimental design described in (a). **(d)** Experimental design of BNT162b2-based cancer therapy in the MB49 bladder cancer model ($n$ = 6 per group). **(e)** Representative

IFN-γ ELISpot shows tumor neoantigen-specific T cell responses post BNT162b2-based cancer therapy. We identified several potential MB49-specific cancer neoantigens with good therapeutic effects (data not shown). **(f)** Quantification of IFN-γ spots in ELISpot assay. **(g)** Experimental design for detecting the antigen spreading induced by BNT162b2-based cancer therapy in a bilateral B16F10 melanoma model ($n$ = 5 per group). **(h)** Photos of the right flank and left flank tumors at the endpoint and tumor growth curves of the intratumoral BNT162b2 treatment group vs. the intratumoral PBS control group in the experiment described in (g).

**Supplementary Fig. S6**

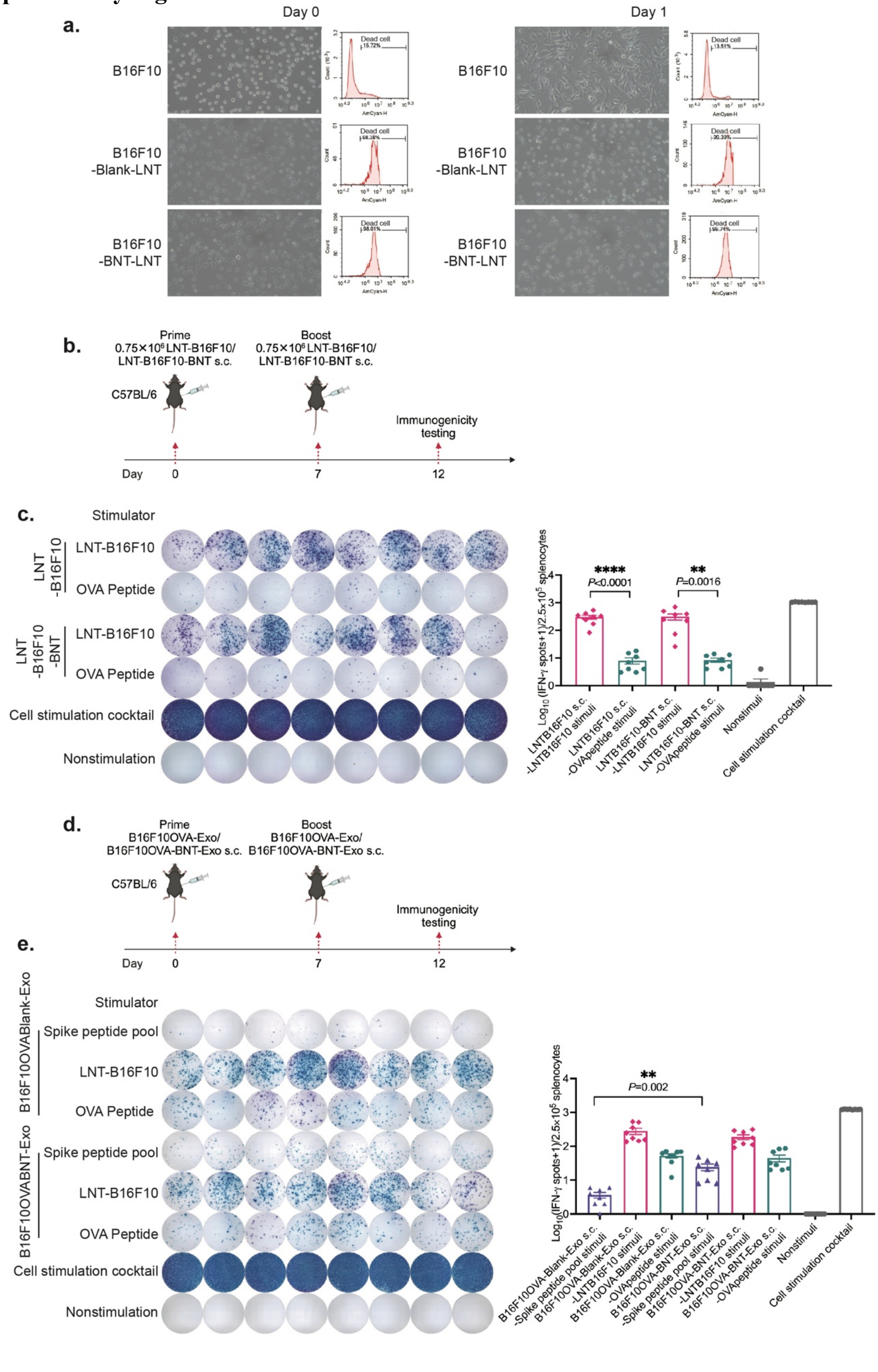

**Supplementary Fig. S6. Immunogenicity of dead tumor cells and tumor cell-derived exosomes.**

**(a)** Cell viability detection of liquid nitrogen-treated (LNT)-B16F10, cell viability was detected by Fixable Viability Dye eFluor 506. **(b)** Experimental design for detecting the immunogenicity of LNT-B16F10 (*n* = 8 per group). **(c)** ELISpot assay results and statistical analysis of the immunogenicity of LNT-B16F10 and LNT-B16F10-BNT (B16F10 got liquid nitrogen treatment after BNT162b2 transfection). **(d)** Experimental design for detecting the immunogenicity of B16F10-OVA-derived exosomes and BNT162b2 transfected B16F10-OVA-derived exosomes (*n* = 8 per group). **(e)** Detection of B16F10-OVA-derived exosomes or BNT162b2 transfected B16F10-OVA-derived exosomes induced T cell responses by ELISpot assay and statistical analysis of the ELISpot assay result.

## Supplementary Fig. S7

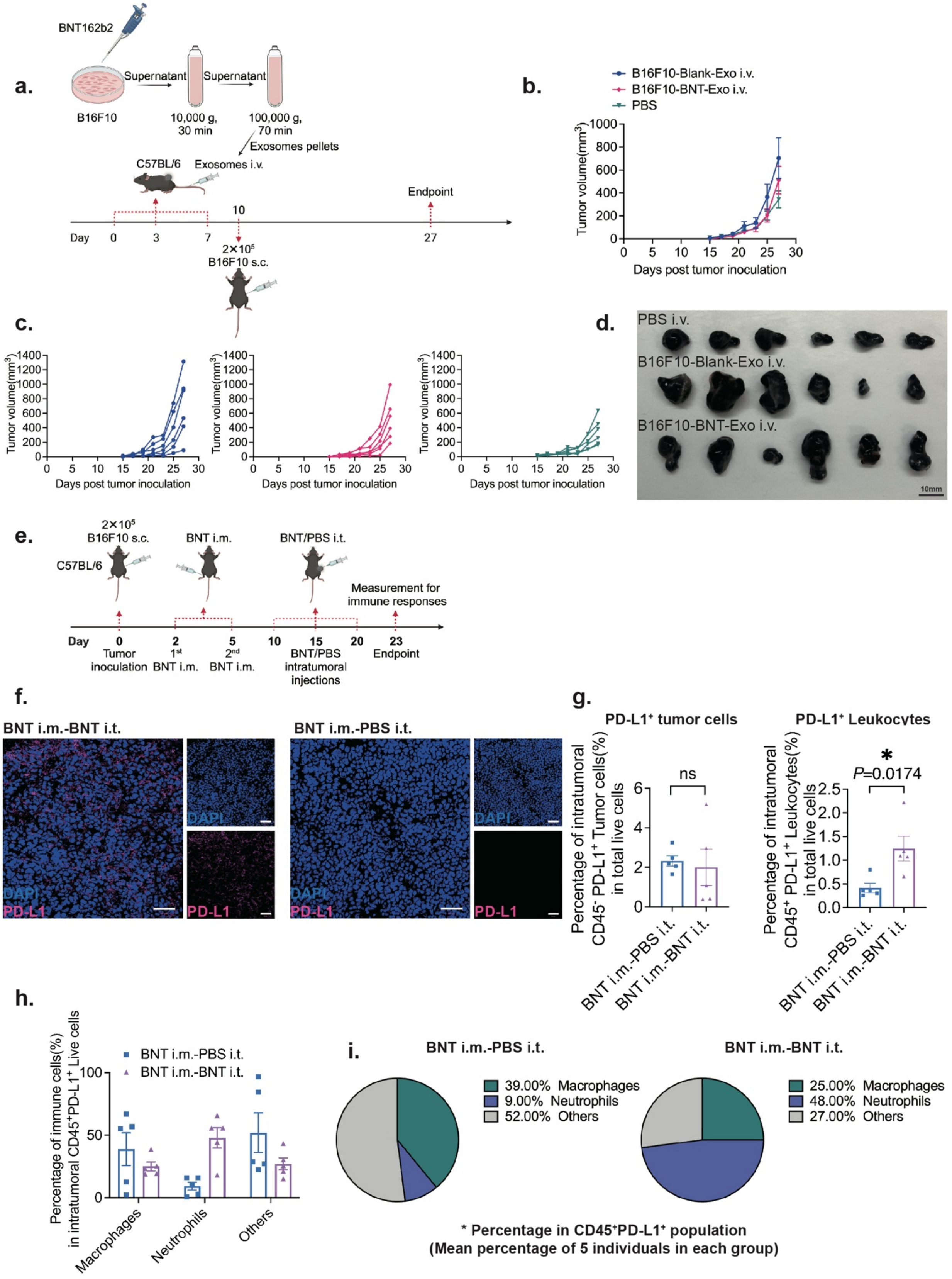

**Supplementary Fig. S7. Increased PD-L1 level in TME post BNT162b2-based cancer therapy.**

**(a)** Experimental design for the investigation of the prevention efficacy of B16F10-derived exosomes and BNT162b2 transfected B16F10-derived exosomes on B16F10 melanoma progression ($n$ = 6 per group). **(b)** Tumor growth curves of the mice received intravenous injections of B16F10-derived exosomes in prevention group *vs*. intravenous injections of BNT162b2 transfected B16F10-derived exosomes in prevention group vs. intravenous injections of PBS in control group for the experiment described in (a). **(c)** Tumor growth curves of the different treatment groups and the PBS control group. **(d)** Photo of tumors from the mice in different treatment groups and the PBS control group at the endpoint of the experiments described in (a). **(e)** Experimental design of the BNT162b2-based cancer therapy. Tumor samples were obtained at the endpoint for immunofluorescent staining (f) and flow cytometry (g - i). **(f)** Representative immunofluorescent staining of PD-L1 on tumor sections from mice described in (e). **(g)** Flow cytometry detection of the percentage of PD-L1$^{+}$ cells in CD45$^{-}$ or CD45$^{+}$ population ($n$ = 5 per group) in tumors from mice described in (e). **(h)** Comparison of the percentage of different types of immune cells in intratumoral CD45$^{+}$PD-L1$^{+}$ live cells between the mice with intratumoral BNT162b2 treatments (BNT i.m.-BNT i.t. group) and the mice with intratumoral PBS treatments (BNT i.m.-PBS i.t. group) ($n$ = 5 per group). **(i)** Analysis of the proportion of intratumoral macrophages (CD11b$^{+}$F4/80$^{+}$) and neutrophils (CD11b$^{+}$F4/80$^{-}$Gr-1$^{+}$) in the CD45$^{+}$PD-L1$^{+}$ population in intratumoral BNT162b2 (BNT i.m.-BNT i.t. group) and intratumoral PBS (BNT i.m.-PBS i.t. group) treatment groups. The percentage of each cell type is shown by the mean of the percentages in five different individuals ($n$ = 5 per group). Scale bar, 50 μm in (f).

**Supplementary Fig. S8**

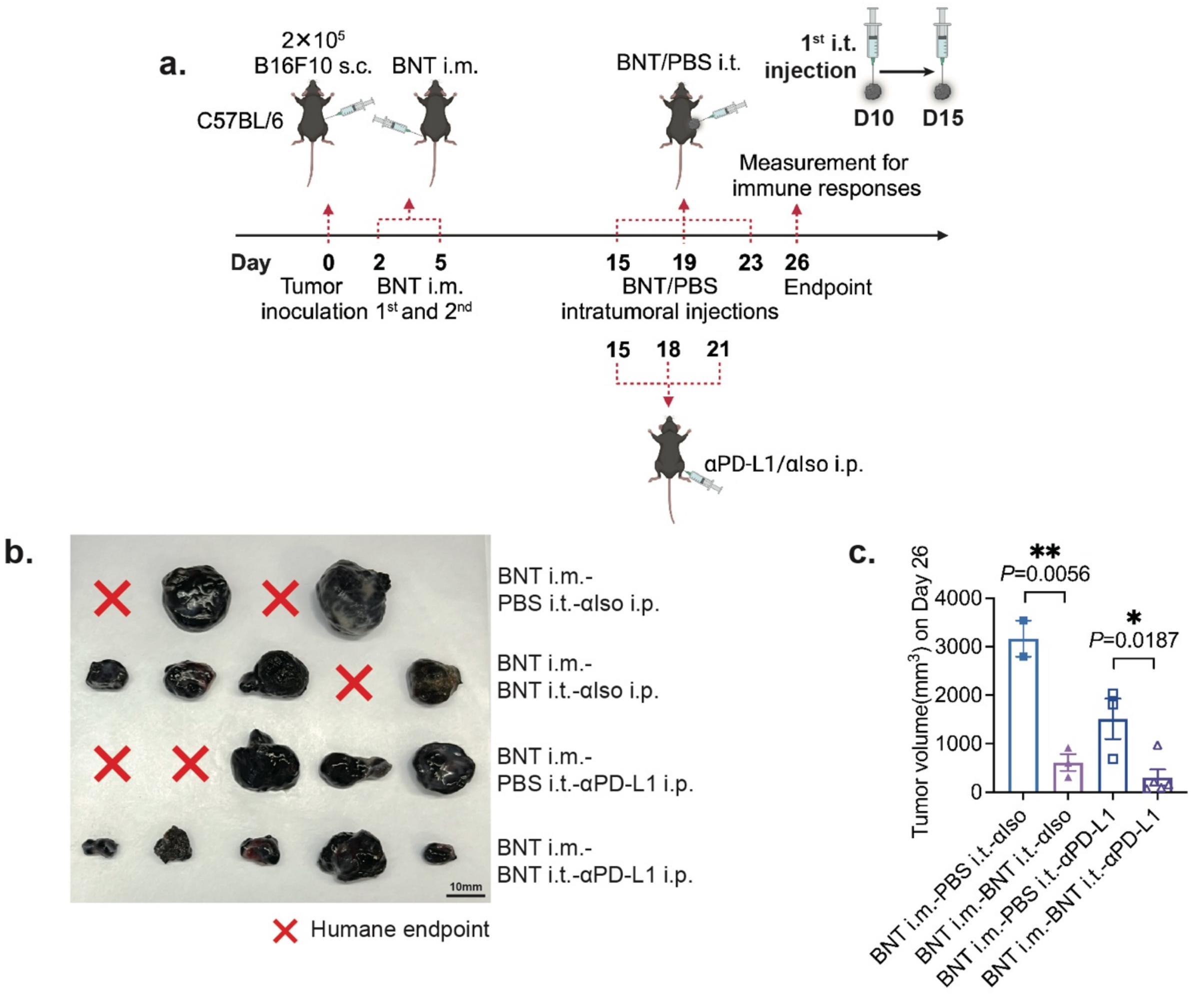


**Supplementary Fig. S8. The combinational therapy of BNT162b2 and anti-PD-L1 effectively enhances the therapeutic efficacy for advanced melanoma.**

**(a)** Experimental design of the BNT162b2-based cancer therapy in the advanced melanoma model. **(b)** Photo of tumors from the mice in the treatment and control groups at the endpoint of the experiments described in (a). **(c)** Tumor volumes of the mice in treatment groups and the control groups for the experiment described in (a). BNT i.m.-PBS i.t.-αIso i.p.: the mice with BNT162b2 intramuscular injections, PBS intratumoral injections, and isotype antibody (αIso) intraperitoneal injections. BNT i.m.-BNT i.t.-αIso i.p.: the mice with BNT162b2 intramuscular

injections, BNT162b2 intratumoral injections, and isotype antibody (αIso) intraperitoneal injections. BNT i.m.-PBS i.t.-αPD-L1 i.p.: the mice with BNT162b2 intramuscular injections, PBS intratumoral injections, and anti-PD-L1 antibody intraperitoneal injections. BNT i.m.-BNT i.t.-αPD-L1 i.p.: the mice with BNT162b2 intramuscular injections, BNT162b2 intratumoral injections, and anti-PD-L1 antibody intraperitoneal injections. The time points of different administrations were shown in (a).

**Table S1. Antibodies for immunofluorescent staining.**

| **Antibodies-Immunofluorescent staining** | | |
|---|---|---|
| **Primary Antibodies** | | |
| **Name** | **Source** | **Cat. No.** |
| SARS Coronavirus Spike Protein Polyclonal Antibody | Thermo Fisher | PA141165 |
| CD3 Antibody-Alexa Fluor 488 | BioLegend | 100210 |
| CD20 Antibody | Thermo Fisher | 16-0201-85 |
| NK1.1 Antibody | BioLegend | 108702 |
| CD11c Antibody-FITC | Thermo Fisher | 11-0114-85 |
| I-A/I-E Antibody | BioLegend | 107602 |
| H-2Kb/H-2Db Antibody | BioLegend | 114602 |
| CD68 Antibody | Thermo Fisher | 14-0681-82 |
| Gr-1 Antibody | Thermo Fisher | 14-5931-85 |
| CD86 Antibody | Thermo Fisher | 14-0862-82 |
| PD-L1 Antibody | BioLegend | 124302 |
| CD63 Antibody | Thermo Fisher | PA5-92370 |
| F4/80 Antibody | Thermo Fisher | MA5-16363 |
| CD206 Antibody | Thermo Fisher | PA5-46994 |
| **Secondary Antibodies** | | |
| **Name** | **Source** | **Cat. No.** |
| Alexa Fluor 488-labeled Goat anti-Rabbit IgG(H+L) Secondary Antibody | Thermo Fisher | A-11008 |
| Alexa Fluor 488-labeled Goat anti-Mouse IgG (H+L) Cross-Adsorbed Secondary Antibody | Thermo Fisher | A-11001 |

| | | |
|---|---|---|
| Alexa Fluor 488-labeled Donkey anti-Rabbit IgG (H+L) Secondary Antibody | Thermo Fisher | A-21206 |
| Alexa Fluor 555-labeled Goat anti-Rabbit IgG (H+L) Cross-Adsorbed Secondary Antibody | Thermo Fisher | A-48283 |
| Alexa Fluor 555-labeled Donkey anti-Goat IgG (H+L) Cross-Adsorbed Secondary Antibody | Thermo Fisher | A-21432 |
| Alexa Fluor 647-labeled Goat anti-Rat IgG (H+L) Cross-Adsorbed Secondary Antibody | Thermo Fisher | A-21247 |
| Alexa Fluor 647-labeled Chicken anti-Rat IgG (H+L) Cross-Adsorbed Secondary Antibody | Thermo Fisher | A-21472 |

**Table S2. Antibodies/Dyes for flow cytometry.**

| **Antibodies/Dyes-Flow cytometry** | | |
|---|---|---|
| **Name** | **Source** | **Cat. No.** |
| TruStain FcX PLUS anti-mouse CD16/32 Antibody | BioLegend | 156604 |
| Brilliant Violet 605-labeled anti-mouse CD45 Antibody | BioLegend | 103140 |
| FITC-labeled anti-mouse CD3 Antibody | BioLegend | 100204 |
| Brilliant Violet 421-labeled anti-mouse CD19 Antibody | BioLegend | 115549 |
| FITC-labeled anti-mouse CD11c Antibody | BioLegend | 117306 |
| Alexa Fluor 700-labeled anti-mouse F4/80 Antibody | BioLegend | 123130 |
| PE-labeled anti-mouse F4/80 Antibody | BioLegend | 123110 |
| APC anti-mouse CD11b Antibody | BioLegend | 101212 |
| Brilliant Violet 421-labeled anti-mouse CD11b Antibody | BioLegend | 101236 |
| Pacific Blue-labeled anti-mouse CD86 Antibody | BioLegend | 105022 |
| PerCP- Cyanine5.5-labeled anti-mouse CD206 Antibody | BioLegend | 141716 |
| PE-labeled anti-mouse I-A/I-E Antibody | BioLegend | 107608 |
| PE/Cyanine7-labeled anti-mouse Ly-6C Antibody | BioLegend | 128018 |
| APC-labeled anti-mouse Ly-6G/Ly-6C (Gr-1) Antibody | BioLegend | 108412 |
| PerCP-Cyanine5.5-labeled anti-mouse PD-L1 Antibody | BioLegend | 124333 |
| Alexa Fluor 700-labeled anti-mouse NK1.1 Antibody | BioLegend | 156512 |
| Brilliant Violet 605-labeled anti-mouse CD69 Antibody | BioLegend | 104530 |
| Fixable Viability Dye eFluor 506 | Thermo Fisher | 65-0866-14 |

| | | |
|---|---|---|
| Fixable Viability Dye eFluor 780 | Thermo Fisher | 65-0865-14 |

**Table S3. Antibodies/Dyes for flow cytometry intracellular staining.**

| **Antibodies/Dyes- Flow cytometry intracellular staining** | | |
|---|---|---|
| **Name** | **Source** | **Cat. No.** |
| Anti-Calreticulin Antibody | Thermo Fisher | MA5-15382 |
| APC-labeled anti-HSP70 Monoclonal Antibody | Thermo Fisher | MA5-45090 |
| APC-labeled anti-mouse CD63 Antibody | Thermo Fisher | 17-0631-82 |
| PE-labeled anti-mouse IFN-γ Antibody | BioLegend | 505808 |
| Fixable Viability Dye eFluor 780 | Thermo Fisher | 65-0865-14 |
| Alexa Fluor 488-labeled Goat anti-Mouse IgG (H+L) Cross-Adsorbed Secondary Antibody | Thermo Fisher | A-11001 |
